# The Precision in Psychiatry (PIP) study: Testing an internet-based methodology for accelerating research in treatment prediction and personalisation

**DOI:** 10.1186/s12888-022-04462-5

**Published:** 2023-01-11

**Authors:** Chi Tak Lee, Jorge Palacios, Derek Richards, Anna K. Hanlon, Kevin Lynch, Siobhan Harty, Nathalie Claus, Lorraine Swords, Veronica O’Keane, Klaas E Stephan, Claire M Gillan

**Affiliations:** 1 School of Psychology, Trinity College Dublin, Dublin, Ireland; 2 Trinity College Institute of Neuroscience, Trinity College Dublin, Dublin, Ireland; 3 SilverCloud Science, SilverCloud Health, Dublin, Ireland; 4 Department of Psychology, Division of Clinical Psychology and Psychological Treatment, Ludwig-Maximilians-University Munich, Munich, Germany; 5 School of Medicine, Trinity College Dublin, Dublin, Ireland; 6 Tallaght Hospital, Trinity Centre for Health Sciences, Tallaght University Hospital, Tallaght, Dublin, Ireland; 7 Institute for Biomedical Engineering, Translational Neuromodeling Unit, University of Zürich & Eidgenössische Technische Hochschule, Zurich, Switzerland; 8 Max Planck Institute for Metabolism Research, Cologne, Germany; 9 Global Brain Health Institute, Trinity College Dublin, Dublin, Ireland

**Keywords:** Treatment response, Treatment outcomes, Treatment prediction, Mental health treatments, Internet-based methodology, Big data, Internet-delivered cognitive behavioural therapy, Antidepressant

## Abstract

**Background:**

Evidence-based treatments for depression exist but not all patients benefit from them. Efforts to develop predictive models that can assist clinicians in allocating treatments are ongoing, but there are major issues with acquiring the volume and breadth of data needed to train these models. We examined the feasibility, tolerability, patient characteristics, and data quality of a novel protocol for internet-based treatment research in psychiatry that may help advance this field.

**Methods:**

A fully internet-based protocol was used to gather repeated observational data from patient cohorts receiving internet-based cognitive behavioural therapy (iCBT) (*N* = 600) or antidepressant medication treatment (*N* = 110). At baseline, participants provided > 600 data points of self-report data, spanning socio-demographics, lifestyle, physical health, clinical and other psychological variables and completed 4 cognitive tests. They were followed weekly and completed another detailed clinical and cognitive assessment at week 4. In this paper, we describe our study design, the demographic and clinical characteristics of participants, their treatment adherence, study retention and compliance, the quality of the data gathered, and qualitative feedback from patients on study design and implementation.

**Results:**

Participant retention was 92% at week 3 and 84% for the final assessment. The relatively short study duration of 4 weeks was sufficient to reveal early treatment effects; there were significant reductions in 11 transdiagnostic psychiatric symptoms assessed, with the largest improvement seen for depression. Most participants (66%) reported being distracted at some point during the study, 11% failed 1 or more attention checks and 3% consumed an intoxicating substance. Data quality was nonetheless high, with near perfect 4-week test retest reliability for self-reported height (ICC = 0.97).

**Conclusions:**

An internet-based methodology can be used efficiently to gather large amounts of detailed patient data during iCBT and antidepressant treatment. Recruitment was rapid, retention was relatively high and data quality was good. This paper provides a template methodology for future internet-based treatment studies, showing that such an approach facilitates data collection at a scale required for machine learning and other data-intensive methods that hope to deliver algorithmic tools that can aid clinical decision-making in psychiatry.

**Supplementary Information:**

The online version contains supplementary material available at 10.1186/s12888-022-04462-5.

## Background

A range of evidence-based treatments for depression exist, including pharmacotherapy, psychological therapies, and neurostimulation. These treatments work on average, but not all patients benefit. In fact, clinical trial data suggests that only 50% of patients respond to the initial treatment they receive, with just 30% achieving remission [1, 2]. Many patients must try multiple, sequential and/or parallel treatments on a trial-and-error basis, each taking weeks or months for potential therapeutic effects to unfold, without guarantee of success [3, 4]. This leads to sustained human suffering, accumulation of side-effects, and substantial economic costs [5, 6].

One potential approach to reducing trial-and-error in psychological treatment is to develop data-informed tools that can assist mental health practitioners in prescribing the best treatment for each individual patient [7]. This type of ‘precision medicine’ approach is not new; for more than two decades, researchers have studied the potential predictive power of a wide range of factors including socio-demographics, clinical characteristics, as well as biomarkers derived from genetic, biochemical, and neuroimaging data [8, 9]. While numerous factors have been observed to have an association with treatment response in individual studies, effect sizes are mostly too small to have real-world clinical value [9, 10].

A solution to this problem may lie in the development of multivariable models that are informed by data from complementary domains, such as cognitive, (neuro)physiological and molecular data [11, 12, 13]. Machine learning is one such method that can iteratively and contemporaneously analyse multiple variables and their interaction, aggregating small individual effects into single predictive values [14]. Using machine learning to optimise treatment approaches is promising, but to-date the published work in depression has suffered from quality issues. A recent review on predicting treatment outcomes in depression highlighted that out of 54 published studies, just 8 met basic quality control standards of including a large sample size (i.e., > 100 participants) and an adequate validation method [15]. For those studies that have large sample sizes, data tend to come from clinical trials that have access to only a small number of variables per patient [15, 16, 17]. This was the case for a model developed by Chekroud and colleagues (2016) that identified 25 self-report demographics and clinical measures which predicted treatment response to antidepressants with 60% accuracy (49% sensitivity and 71% specificity) in their held-out test dataset [18]. Subsequently, Iniesta and colleagues (2018) trained an algorithm using a combination of clinical and molecular genetic data, which achieved high predictive performance (0.77 in the area under the receiver operating characteristic curve) in external data (69% sensitivity and 71% specificity) [19]. This suggests that incorporating different data modalities may be an important next step for improving the performance of these models.

One way to acquire these datasets is through multi-site collaboration via research consortia such as the EMBARC [20], PReDICT [21] and iSPOT-D [22] studies. These large randomised controlled trials are gold-standard, but are costly, time-consuming, resource intensive, and due to the involvement of many sites, are logistically complex. Therefore, there is a growing need to find alternative methodologies that can complement these approaches, providing us with larger datasets, more rapidly, and in more diverse populations.

To address these gaps, the Precision in Psychiatry (PIP) study used a novel internet-based protocol to recruit, comprehensively assess, and follow through time, mental health patients about to initiate internet-delivered cognitive behavioural therapy or antidepressant medication. Here, we tested the feasibility of this internet-based methodology in collecting large-scale patient data of various types, at home and in a flexible manner. We outline in detail the design of this study, the patient demographic and clinical characteristics, pre-post clinical changes, study attrition, schedule compliance, treatment adherence, data quality, and qualitative patient-perspectives gathered from exit surveys. In examining these facets of the study, we aim to provide guidance for the design of future internet-based studies by highlighting which factors favourably influence recruitment and data collection. We discuss the benefits and limitations of this methodology and make suggestions for future studies adopting a similar approach.

## Methods

### Participant Identification and Recruitment

*Internet-delivered Cognitive Behavioural Therapy (iCBT).* Participants receiving clinician-guided iCBT on the SilverCloud Health platform were digitally recruited from two sites: (i) a National Health Service (NHS) mental health service ‘Talking Therapies’ based near Reading, West London, United Kingdom (*Berkshire Foundation Trust*) and (ii) a mental health charity based in Dublin, Ireland (*Aware Ireland*) that provides free education programs, and information services for the public impacted by mood-related conditions. A key difference across sites was that at Talking Therapies, patients have an initial consultation with a clinician who assesses the patient’s needs before deciding whether to offer them an iCBT program via SilverCloud. In contrast, individuals recruited via the Aware charity are self-referring. At both sites, the iCBT intervention includes clinician support via the platform. At Berkshire, clinicians are made up of specially trained psychology graduates called Psychological Wellbeing Practitioners (PWPs). At Aware, graduate volunteers provide the clinical support. All supporters have been trained in using the platform. Potential participants at each site received an automated ‘Welcome’ email upon registering for SilverCloud, which contained an invitation to participate in this study via a web-link.

*Antidepressant Medication.* Individuals initiating antidepressant medication were recruited internationally using a combination of online (Google Ads, social media platforms, mental health charities) and in-print advertisement campaigns (pharmacies, general practitioners, counselling clinics, newsletters). Participants in the antidepressant arm were asked to provide details on the type and dosage of the antidepressant medication treatment they were prescribed, and to upload a photograph of their prescription for verification purposes. Participants were not required to be medication-free prior to starting the study. They were eligible to participate if they were about to experience a change in pharmacotherapy; initiating, switching, or adding medication.

### Screening and Study Entry Requirements

*Screening.* In both treatment arms, participants read the information sheet online and provided electronic consent. Participants were notified that their participation was entirely voluntary and would not impact on their care in any way, and that their clinician would not be notified about their participation. They were also informed that they were free to terminate or alter their treatment at any time during the study, and that this would not affect their ability to continue to participate and receive payment. After providing informed consent, participants in both arms were directed to a screening survey used to determine their eligibility. Participants provided their age, English language fluency, email address, listed medications they were taking, confirmed computer access, and told us where they heard about the study. Participants indicated whether they had already started treatment, or if they were planning to start in the future and provided an approximate treatment start date. As stated above, participants in the antidepressant arm also provided a photo of their prescription, which was manually checked for drug name, dose, and date prior to their admission. All participants completed the Work and Social Adjustment Scale (WSAS) [23], which was used to determine eligibility.

*Inclusion/Exclusion Criteria. *Participants in both arms were excluded if they were not between 18–70 years of age, were not fluent in English, or reported that they did not have computer access. Participants were also required to score a 10 or above on the WSAS [23] which is a transdiagnostic measure capturing impairments in daily functioning arising from mental health problems. In the antidepressant arm, participants were required to have recently started (< 2 days ago) or be planning to start/change treatment soon (< 2 days from now). If they indicated that they were planning to start their treatment in > 2 days after the study sign-up date, they were contacted via email and advised to reapply for the study closer to their treatment start date. In the iCBT arm, participants were invited to our study via automated email directly following their registration on the SilverCloud platform. Given the self-paced nature of iCBT (i.e., users undertake the treatment at their own pace, and can freely choose the order of intervention modules and content they complete in), we also asked participants to indicate when they planned to start treatment. Participants who indicated that they had already started iCBT > 2 days prior to signing up were not included in the study, and those who indicated that they planned to start in > 2 days were contacted via email, and a treatment start date and study schedule agreed upon with the research team manually. In those cases, patients had technically registered on the platform, but were not planning to engage in the modules immediately for various personal reasons.

### Study Schedule

**Fig. 1:**
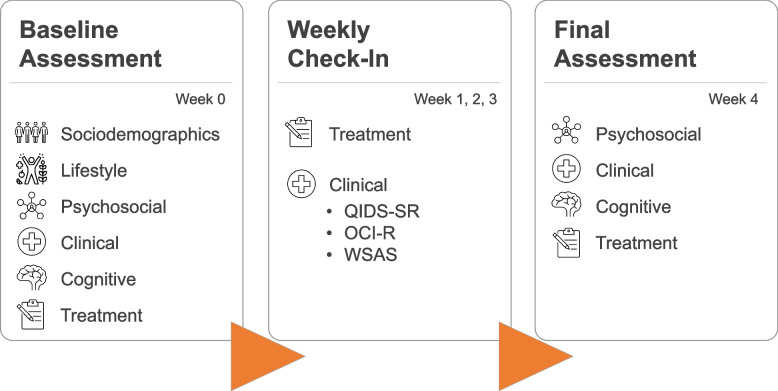
An overview of study design. Participants who gave informed consent and met our inclusion/exclusion criteria were invited to complete the baseline assessment, comprising cognitive tests, and a variety of self-report questions concerning participants’ treatment, clinical symptoms, psychosocial factors, lifestyle, and socio-demographics. Participants were sent an invitation for a weekly check-in assessment on a scheduled basis for 3 consecutive weeks, which tracked any changes in clinical symptoms and treatment adherence. Participants completed the study with a fifth and final assessment after 4 weeks of treatment, which was an abbreviated version of the baseline assessment

If participants were deemed eligible to take part in the study, they were sent an individualised study schedule and a web-link for completing the baseline assessment. While we endeavoured to have participants complete the baseline on the same day they initiated treatment, we took a pragmatic and flexible approach, allowing participants a window of 4 days from their treatment start date in which to complete the baseline assessment. Four participants in the antidepressant arm completed their baseline assessments 5 days after their treatment start date due in part to administrative issues, and we chose to retain their data for analysis. In the iCBT arm, there were no participants outside of this criterion. Weekly check-in assessments and the final assessment for each participant were approximately scheduled at a 7-day interval following their treatment start date and were provided to participants 1 day before they were due with the instruction to complete them on the following day. Figure 1 shows an overview of the study design and the assessments involved at each timepoint.

### Outcome Measure

The primary outcome measure for this study is percent change in depression symptom severity on the Quick Inventory of Depressive Symptomatology – Self-Report (QIDS-SR) [24]. For certain types of planned machine learning analysis that require categorical outcomes, we binarize this as ‘early response’, defined as ≥ 30% pre-post improvement. We selected this because (i) participants are not expected to achieve ‘response’ (i.e., ≥ 50% pre-post improvement in QIDS-SR) or ‘remission’ (i.e., QIDS-SR score of ≤ 5) in a 4-week timeframe and (ii) prior work has shown that this threshold of early response is a strong indicator of 8-week clinical outcomes [25].

### Baseline Assessment

The baseline assessment took approximately 1.5–2 hours to complete and required a mouse and a keyboard. Six categories of data were gathered, spanning (i) clinical data, (ii) treatment data, (iii) cognitive test data, (iv) socio-demographics, (v) psychosocial factors and (vi) lifestyle factors (see Additional File 2 – Variable Directory for a full outline of variables collected in the study).

*Clinical Data. *To assess whether treatment has a transdiagnostic effect on mental health, we considered the WSAS as a secondary outcome, measuring general impairment in psychosocial functioning due to mental health problems [23]. To assess specific clinical changes, we administered a range of clinical self-report scales assessing obsessive–compulsive disorder measured by the Obsessive–Compulsive Inventory – Revised (OCI-R) [26], depression measured by the Self-Rating Depression Scale (SDS) [27], trait anxiety measured by the trait portion of the State-Trait Anxiety Inventory (STAI) [28], alcohol addiction measured by the Alcohol Use Disorder Identification Test (AUDIT) [29], apathy measured by the Apathy Evaluation Scale (AES) [30], eating disorders measured by the Eating Attitude Test (EAT-26) [31], impulsivity measured by the Barratt Impulsivity Scale (BIS-10) [32], schizotypy measured by the Short Scales for Measuring Schizotypy (SSMS) [33], and social anxiety measured by the Liebowitz Social Anxiety Scale (LSAS) [34]. These instruments allow for the estimation of 3 transdiagnostic dimensions (anxious-depression, compulsivity, and social withdrawal) based on factor loadings identified in a prior study [35]. These transdiagnostic dimensions have been shown to map onto certain aspects of cognition better than standard questionnaires, such as model-based planning [35, 36] and metacognitive bias [37, 38]. In addition to self-report symptoms, we assessed our participants’ history and chronicity of mental health problems. More specifically, we assessed the number of mental health episodes they have experienced, what age they were when they experienced their first mental health episode, the duration of their current mental health episode, the number of psychiatric diagnoses they had, and the number of close family members with psychiatric diagnoses. As previously mentioned, Chekroud and colleagues’ study (2016) developed a predictive model that achieved ~ 60% accuracy in predicting antidepressant response in a re-analysis of the Sequenced Treatment Alternatives to Relieve Depression (STAR*D) dataset [18]. We further included 8 miscellaneous items from this study in order to recapitulate their model as a benchmark against which to compare our own (see Additional File 2 – Variable Directory).

*Treatment Variables. *Treatment variables included history of medication and/or psychological treatments for mental health, concurrent medication and/or psychological treatments for mental health, as well as participants’ expectations about the mental health treatment they were about to initiate. For participants in the iCBT treatment arm, we examined objective engagement data for each participant, which was provided by SilverCloud.

*Cognitive Test Data. *Participants completed 4 browser-based gamified cognitive tasks in randomised order, interspersed with blocks of self-report assessments as outlined in the previous section. These were implemented in JavaScript and Python, hosted on a server at Trinity College Dublin and were accessible through any commonly used web-browser. Participants completed a two-step decision making task [39, 40] which estimates various reinforcement learning parameters, including separate estimates of model-based and model-free learning, choice perseveration, and learning rate. Prior studies have shown that model-based planning is linked to compulsivity in the general population [35] and compulsive disorders like obsessive–compulsive disorders (OCD) [41], which benefit from antidepressant medication (albeit at higher doses) [42] and are commonly co-morbid with anxiety and depression [43]. The second task in our battery is an aversive learning task that manipulates environmental volatility [44] to assess the extent to which participants adjust their learning rate appropriately as volatility increases. A reduced sensitivity to volatility has been previously linked to trait anxiety and the functioning of the noradrenergic system [45]. The third task we included measures metacognitive bias and sensitivity in the context of perceptual decision making. Individuals who score high on a transdiagnostic dimension of anxious-depression symptoms have lower confidence in their decision-making, while those high in compulsivity have over-confidence [36, 37]. Our final cognitive assessment was abstract reasoning using a computerised adaptive task based on Raven’s Standard Progressive Matrices [46]. Reasoning deficits are associated with risk for various mental health conditions [47].

*Socio-Demographics. *In addition to age, which they reported at study intake, participants self-reported their sex, country of residence, marital status, education level, subjective social status, and employment status.

*Psychosocial Variables. *Perceived social support was assessed using the Multidimensional Scale of Perceived Social Support (MSPSS) [48], perceived stress was assessed using the Perceived Stress Scale (PSS) [49], experience of stressful life events in the past 12 months was assessed using the Social Readjustment Rating Scale (SRRS) [50], and childhood traumatic experiences were measured by the Childhood Trauma Questionnaire (CTQ) [51].

*Physical Health and Lifestyle. *This included exercising habits, smoking habits, dietary quality, current and prior recreational drug use, height, and weight. Physical health comorbidities were measured by the Cumulative Illness Rating Scale (CIRS) [52], and somatic symptoms were measured by 5 items pertaining to stomach, back, limbs, head, and chest pain in the Patient Health Questionnaire-15 (PHQ-15) [53].

### Weekly Check-Ins

Weekly check-in assessments were sent to participants in each week of the study. They could be completed using a computer, tablet, or smartphone and took approximately 10-15 minutes to complete. Participants had 4 days to complete these assessments or were otherwise excluded from further participation. They completed 3 standardised questionnaires each week, including the QIDS-SR for depression symptoms, the WSAS for impairment symptoms, and the OCI-R for OCD symptoms. In addition, participants also answered questions about treatment adherence, side effects and dosage changes (for those in the antidepressant arm), whether they initiated any other mental health treatments, and other extra relevant information they wished to inform the study co-ordinators after they have begun participation in the study.

### Final Assessment

Participants were asked to complete a detailed final assessment after 4 weeks of treatment. This was almost identical to the baseline assessment, comprising 4 gamified cognitive tasks and self-report questionnaires administrated in a randomised order. Self-report variables gathered during the baseline assessment that were not expected to change (e.g., childhood trauma, age, education etc.) were not re-collected (see Table S1 in Additional File 1 – Supplementary Materials for schedule of assessments). Contingent on completion of the final assessment, a proportion of participants were invited to complete a short feedback survey on their experience of the study and to provide suggestions for future studies with similar scope and design.

### Quality Control

Participants completed their assessments in an at-home environment where traditional experimental control is absent. To understand how this might affect data quality, we included questions to help us identify bad quality data. At the end of both the baseline and final assessments, participants were asked if they were distracted during the session and if so, by what. They were also asked if they had consumed any substances (e.g., alcohol/drugs) 5 hours prior to participation. Participants were assured their continued participation would not be affected by their response. In addition, we included a ‘catch question’ that was embedded in both the OCI-R and WSAS questionnaires at baseline and in the WSAS questionnaire at all subsequent timepoints. These 6 catch questions asked participants to select a specific answer option if they were paying attention.

### Clinical Interventions

*Internet-Delivered Cognitive Behavioural Therapy (iCBT). *SilverCloud provides low-intensity, clinician-guided iCBT intervention programs for a range of common mental health problems (e.g., ‘Space from Depression’, ‘Space from Anxiety’, ‘Life Skills’, ‘Space from Stress’). The programs partially overlap in terms of content, but also have unique components. All follow evidence-based cognitive behavioural therapy (CBT) principles [54, 55]. Each module takes approximately 1 hour to complete, and while users can self-pace, they are generally recommended to complete at least 1 module per week. The intervention comprises cognitive, emotional, and behavioural components (e.g., behavioural activation, self-monitoring, activity scheduling, mood, and lifestyle monitoring). Each module incorporates introductory quizzes and videos, interactive activities, informational content, as well as homework assignments and summaries. Personal stories and accounts from other users are also included into the presentation of the content. The interventions additionally provide tailored content and modules dependent on the user’s clinical presentation (e.g., ‘Challenging Core Beliefs’ module for depressive symptoms; ‘Worry Tree’ activity for managing symptoms of anxiety). Although the programs are clinician-guided, users are welcome to engage with the modules and content at their own pace and in the order they opt. A clinician, typically an Assistant Psychologist or a Psychological Wellbeing Practitioner [56] trained in the delivery of SilverCloud iCBT programs, is assigned to a user once they have registered and guides their progress through the intervention. During treatment, the clinician reviews the user’s progress while leaving feedback and responding to queries. Typically, 6-8 weekly/fortnightly review sessions are offered across the supported period of the intervention (up to 12 weeks), however, this depends on the user’s specific needs.

*Antidepressant Medication. *Participants in the antidepressant group (*N* = 92) were initiating a range of antidepressant medications. Most (86%) were taking selective serotonin reuptake inhibitors (SSRIs), but 13% were taking serotonin-norepinephrine reuptake inhibitors (SNRIs), 7% taking atypical antidepressants, and 2% were taking tricyclic antidepressants (TCAs). Due to polypharmacy, these numbers do not add to 100%. 8% of participants were taking more than 1 antidepressant medication and 5% were taking another non-antidepressant medication. The most common antidepressant medications were Sertraline (40%), Escitalopram (19%), and Fluoxetine (15%) (see Table S2, S3, and S4 in Additional File 1 – Supplementary Materials). Most participants (90%) experienced side effects from their treatment, with the most common including sleep-related problems such as day-time sleepiness (59%) and night-time sleep disturbances (55%), gastrointestinal symptoms (52%), migraines and headaches (36%), and sexual problems (36%).

### Compensation

Participants in both arms were paid €60 in an accelerating payment schedule through PayPal or digital gift cards. Participants received €10 for completing the baseline assessment, €20 euros after the third weekly check-in, and €30 upon completion of the final assessment. The feedback survey was optional and compensated with an additional €10.

### Data Analysis

In this paper, data are reported on participants who have fully completed the study in the iCBT and antidepressant arms, recruited from 4^th^ February 2019 to 20^th^ July 2021 (*N* = 594). Where appropriate, participants’ recruitment trajectory, socio-demographic and clinical characteristics, treatment, and study compliance data (e.g., retention rates) were compared between study arms using chi-square, t-tests, and repeated measures analyses of variance (ANOVA) (for comprehensive results see section ‘Between-Group Comparisons’ in Additional File 1 – Supplementary Materials). The significance of results (p-value) is reported for context and comparison, however, this study is largely descriptive in nature rather than focusing on hypothesis testing. As such, no correction for multiple comparisons was conducted. To assess data quality, we reported on the numbers of participants who were inattentive, distracted or intoxicated; and to assess the impact of this on data quality, we compared response consistency (i.e., the correlation of similar self-reported items) across the groups who were and were not flagged by these criteria. Finally, a qualitative content analysis was conducted on 4 open-ended free-text questions from the online feedback survey. This method allows researchers to quantify concepts in the data by counting the number of times these concepts appeared, thus providing descriptive statistics fit for the quantitative reporting of this data [57].

## Results

### Participant Recruitment and Retention

**Fig. 2:**
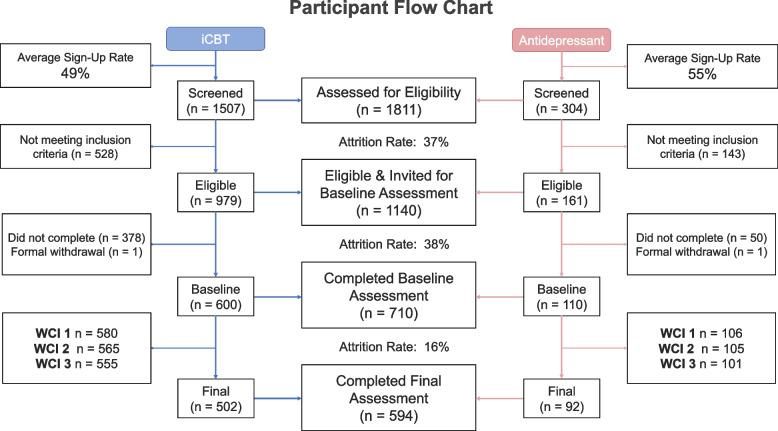
Participant flow chart (CONSORT chart). Once they completed the assessment at each study timepoint, participants were progressed onto the next stage of the study. Participants were progressed if they completed the assessments fully at each study stage. If due to technical errors participants were not able to complete specific components of their assessments, it was deemed appropriate to progress them onto the next stage of the study or be financially compensated

**Fig. 3:**
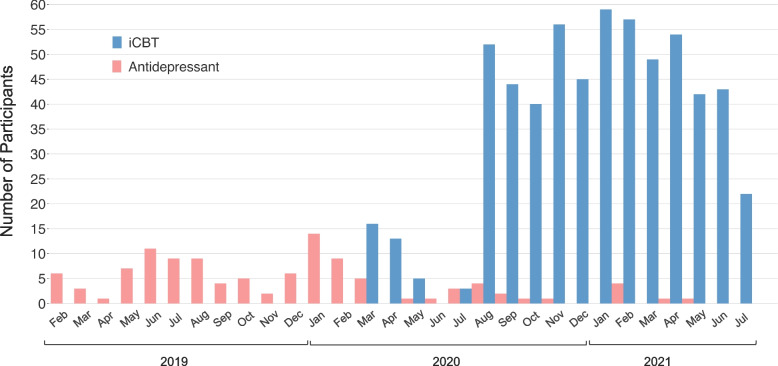
Recruitment Rates. Number of participants recruited from each arm from February 2019 to July 2021. The antidepressant arm launched first, initiating recruitment in February 2019. Paid recruitment efforts were focused on a 13-month period from that date to March 2020, when the iCBT arm commenced. The iCBT arm was initiated in March 2020 via Aware Ireland, and in August 2020 recruitment began through Talking Therapies, Berkshire, South London, U.K

Detailed information regarding recruitment and retention are presented in Fig. 2. Recruitment for the antidepressant arm began in February 2019 and the iCBT arm began in March 2020.^1^ For the iCBT arm, once both sites were active (Aware and Berkshire), we reached a peak recruitment rate of 59 per month, with a mean of 47 (SD = 10.14) (estimated for 12 months, August 2020—July 2021). In the antidepressant arm, active paid and unpaid recruitment via multiple sources spanned February 2019 to March 2020 (13 months), with a peak of 15 per month and a mean of 7 (SD = 3.67). The arm remained open for participants after that time, but active advertising efforts were halted (Fig. 3). At the time of article preparation, screening data from *N* = 1811 were assessed for eligibility across the iCBT (*N* = 1507) and antidepressant (*N* = 304) arms. Of those eligible participants, 63% of participants completed the baseline assessment (*N* = 710), comprising *N* = 600 in the iCBT arm and *N* = 110 in the antidepressant arm. For both groups, retention of baseline completers to weekly check-in 3 was excellent at ≥ 92%, only dropping to 84% for the final assessment. For study completers in the antidepressant arm, most were referred from Google Ads (39%), followed by advertisements through pharmacies (25%), social media campaigns (11%), and general practitioners (8%). For the iCBT group, all were referred from SilverCloud and most came from Talking Therapies in the United Kingdom (83%) and the remaining through Aware Ireland (17%).

### Patient Demographic and Clinical Characteristics

Baseline characteristics of the study completers in both treatment arms are presented in detail in Tables 1 and 2 (see Table S5 and S6 in Additional File 1 – Supplementary Materials for baseline characteristics of baseline completers). Participants in both arms were primarily young in their mid- to late-twenties, white, female, employed, third level educated, came from the United Kingdom and Ireland, and subjectively rated themselves on average in the middle of social class status. Most participants reported having 1 or more mental health diagnoses, the most common being depression and/or generalised anxiety. Most participants reported not having a family member with mental health illness, but they themselves have had ≥ 2 lifetime mental health episodes which first began in their adolescence/adulthood. Most participants reported not having engaged in mental health treatment before, and in their self-report of expectations about treatment efficacy on a scale from 0–9 (“I don’t expect to feel any better” to “I expect to feel completely better”), the average patient rated a 5.

**Table 1:** Baseline demographic characteristics

**Sample Characteristics**	iCBT			Antidepressant			t / *X*^2^ (df)	*p*
	N	%	Median (SD)	N	%	Median (SD)		
**Sex**	502			92			4.21 (3)	0.24
Female	391	77.89		65	70.65			
Male	107	21.31		25	27.17			
Other	4	0.80		2	2.17			
**Country**	499			94			86.93 (2)	< 0.001
UK	407	81.56		38	41.30			
Ireland	84	16.83		38	41.30			
Other	8	1.60		16	17.39			
**Age**	501		29 (11.10)	91		26 (9.98)	-1.78 (590)	0.08
**Marital Status**	502			94			1.39 (5)	0.93
Single	191	38.05		39	42.39			
In a Relationship	150	29.88		28	30.43			
Married	128	25.50		19	20.65			
Divorced	18	3.59		3	3.26			
Separated	14	2.79		3	3.26			
Widowed	1	0.20		0	0.00			
**Education Level**	502			94			3.64 (2)	0.16
< Third Level	122	24.30		14	15.22			
Some/Complete Third Level	268	53.39		55	59.78			
> Third Level	112	22.31		23	25.00			
**Employment Status**	502			94			12.81 (2)	0.002
Employed	346	68.92		46	50.00			
Unemployed	150	29.88		45	48.91			
Retired	6	1.20		1	1.09			
**Subjective Social Status**^**a**^	502		4 (1.68)	92		4 (2.06)	1.03 (592)	0.30

^a^Subjective Social Status is measured by the MacArthur Scale of Subjective Social Status (i.e., the SES ladder) [58]. The scale has a range of 0–10, where the higher the score, the higher the rating of subjective social status

Outliers were not excluded in the descriptive analyses of demographic characteristics

**Table 2:** Baseline clinical characteristics

**Sample Characteristics**	iCBT			Antidepressant				*t* / *X*^2^ (df)	*p*
	N	%	Median (SD)	N	%	Median (SD)			
**No. of Current Diagnosis**	502			92				21.67 (2)	< 0.001
None	155	30.88		8	8.70				
One	183	36.45		37	40.22				
> One	164	32.67		47	51.09				
**Types of Diagnoses**^**a**^	502			92				9.57 (5)	0.09
None	155	30.88		8	8.70				
Depression	245	48.80		70	76.09				
GAD	209	41.63		48	52.17				
Panic Disorder	25	4.98		4	4.35				
PTSD	20	3.98		13	14.13				
OCD	23	4.58		4	4.35				
Others	41	8.17		13	14.13				
**Family with Mental Disorders**	502			92				1.80 (3)	0.62
None	207	41.24		32	34.78				
One	156	31.08		30	32.61				
Two	81	16.14		16	17.39				
≥ Three	58	11.55		14	15.22				
**No. of Lifetime Episodes**	494			91				11.09 (2)	0.004
< 2	53	10.73		7	7.69				
2–5	246	49.80		31	34.07				
> 5	195	39.47		53	58.24				
**Age of onset (years)**	492			92				8.68 (2)	0.01
Childhood (1–12)	86	17.48		23	24.47				
Teenage (13–17)	211	42.89		47	50.00				
Adulthood (18–70)	195	39.63		22	23.40				
**Current episode length (days)**	457		199 (2557)	84			190 (2463)	0.05 (539)	0.96
**History of Past Treatment**	502			92				5.95 (3)	0.11
Never Before	224	44.62		30	31.91				
Psychotherapy & Medication	115	22.91		28	29.79				
Medication only	82	16.33		14	14.89				
Psychotherapy only	81	16.14		20	21.28				
**Treatment Expectation (0–9)**	502		5 (2.04)	92			5 (1.89)	-1.95 (592)	0.05

^a^Types of Diagnoses: The total number of diagnoses type exceeds the sample size of baseline completers (i.e., participants have the option to pick more than one diagnosis)

Outliers were not excluded in the descriptive analyses of clinical characteristics

**Fig. 4:**
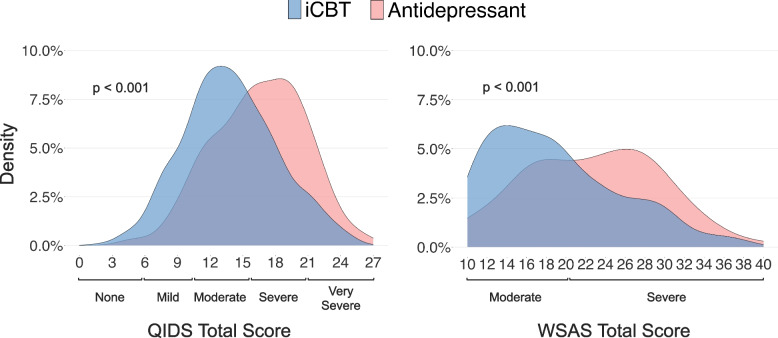
Baseline clinical symptom score distribution of depression (QIDS) and impairment (WSAS) for participants in the iCBT and antidepressant arm

In terms of clinical severity at baseline, participants in the antidepressant arm had a mean QIDS-SR score of 16.51 (SD = 4.17) and a mean WSAS score of 22.73 (SD = 6.84), indicating severe depression [24] and functioning [23], respectively. Participants in the iCBT arm had a somewhat lower QIDS-SR score of 13.86 (SD = 4.28) at baseline, corresponding to moderate depression severity, and a mean WSAS score of 19.02 (SD = 6.65), also falling in the moderate range (Fig. 4). Clinical severity of other symptoms assessed at baseline are presented in Table 3 and Figure S1.

**Table 3:** Baseline clinical symptom scores across treatment arms

**Clinical Symptoms**	iCBT		Antidepressant		t (df)	*p*
	Mean	SD	Mean	SD		
Depression (QIDS-SR)	13.86	4.28	16.51	4.17	5.47 (592)	< 0.001
Impairment (WSAS)^a^	19.02	6.65	22.73	6.84	4.61 (580)	< 0.001
Apathy (AES)	41.89	8.94	43.31	9.46	1.49 (592)	0.14
Alcohol Use (AUDIT)^a^	5.82	5.57	7.74	7.78	2.82 (589)	0.005
Impulsivity (BIS)	67.50	11.15	68.21	10.88	0.56 (592)	0.57
Eating Disorder (EAT)	12.15	10.83	13.99	12.38	1.46 (592)	0.14
Social Anxiety (LSAS)	38.45	16.62	40.89	17.14	1.29 (592)	0.20
OCD (OCI-R)	23.45	12.64	25.38	12.87	1.34 (592)	0.18
Schizotypy (SSMS)	19.09	6.87	22.10	7.50	3.81 (592)	< 0.001
Depression (SDS)	54.17	8.09	58.55	7.91	4.80 (592)	< 0.001
Trait Anxiety (STAI)	61.13	8.78	65.08	8.66	3.97 (592)	< 0.001

^a^At baseline, *N* = 3 were missing AUDIT symptom score and *N* = 12 were missing WSAS symptom score

### Pre-Post 4-Week Clinical Changes

**Fig. 5:**
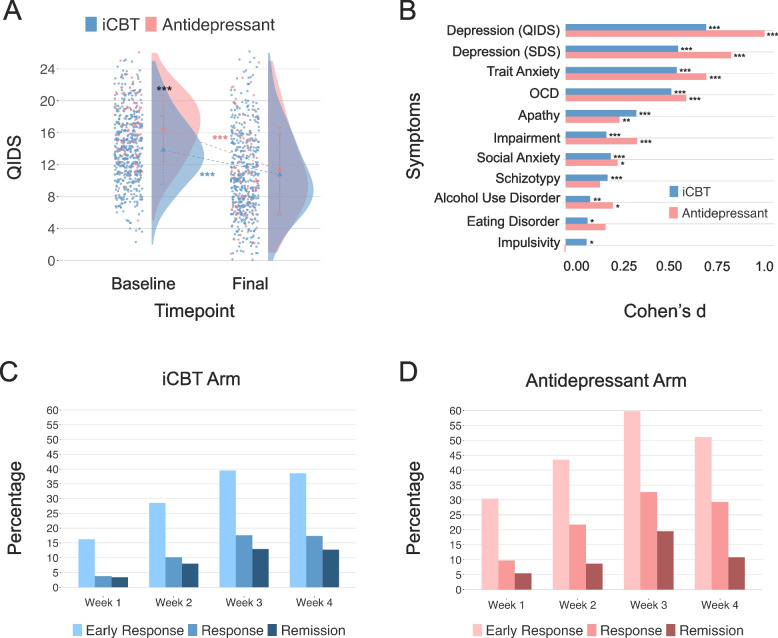
Clinical change in QIDS-SR. (**A**) Pre-post 4-week QIDS-SR score reduction. Both treatment arms experienced significant decreases in depression score measured by QIDS-SR from the baseline to the final assessment. (**B**) Effect sizes and statistical significance of clinical symptom reduction in both treatment arms. All clinical symptoms reduced significantly from the baseline to final assessment in both treatment arms except for schizotypy, eating disorder symptoms, and impulsivity in the antidepressant arm. (**C**) Percentages of early response, response, and remission achieved by participants in the iCBT arm at each study timepoint. (**D**) Percentages of early response, response, and remission achieved by participants in the antidepressant arm at each study timepoint

Participants in both arms experienced significant improvement in depression symptoms after 4 weeks of treatment. Participants in the antidepressant arm experienced a significantly larger percent reduction in QIDS-SR from baseline than those in the iCBT arm, t(589) = 2.73, *p* = 0.007, even after controlling for imbalances in baseline severity, F(1, 588) = 4.36, *p* = 0.04.^2^ Figure 5C, D show the weekly percentage distribution of participants achieving early response, response, and remission throughout the study for each of the two treatment arms. For the iCBT arm, by week 4, 39% of participants have achieved early response (i.e., a 30% reduction), 17% of participants have achieved response (i.e., a 50% reduction) and 13% of participants have achieved remission (i.e., a score ≤ 5). Participants in the antidepressant arm exhibited a significantly higher rate of early response at 51%, *X*^2^ = 5.09 (1), *p* = 0.02, as well as rate of response at 29%, *X*^2^ = 7.19 (1), *p* = 0.007, but no significant difference in their remission rate of 11%, *X*^2^ = 0.24 (1), *p* = 0.62. In terms of absolute score change, in the iCBT arm, QIDS-SR depression scores were significantly reduced by an average of 3.09 points (SD = 4.31) (21%), t(500) = 16.06, *p* < 0.001, with a moderate effect size, *d* = 0.72. In the antidepressant arm, this reduction was larger with an average of 5.18 points (SD = 5.11) (31%) on the QIDS-SR, t(91) = 9.73, *p* < 0.001, with a large effect size, *d* = 1.01. A two-way ANOVA confirmed this difference was significant, F(1, 591) = 17.26, *p* < 0.001 (Fig. 5A).

In terms of general functional impairment (WSAS), there were modest but significant improvements in both treatment groups. Participants in the iCBT arm saw their self-reported impairment reduce by 1.57 points (SD = 7.55) (8%), t(499) = 4.65, *p* < 0.001, *d* = 0.21 and those in the antidepressant arm reported reductions of 3.09 points (SD = 8.46) (14%), t(79) = 3.27, *p* = 0.002, *d* = 0.37. These percentage changes did not differ significantly across the treatment arms, t(572) = 1.36, *p* = 0.17.^3^ Consistent with a transdiagnostic perspective on mental health, clinical gains extended beyond depression symptoms and daily functioning. Analyses revealed significant reductions in most clinical symptoms gathered in both treatment arms (all *p* < 0.05), except for schizotypy (*p* = 0.10), impulsivity (*p* = 0.95), and eating disorder symptoms (*p* = 0.05) in the antidepressant arm (Fig. 5B).

### Study Schedule Compliance

**Fig. 6:**
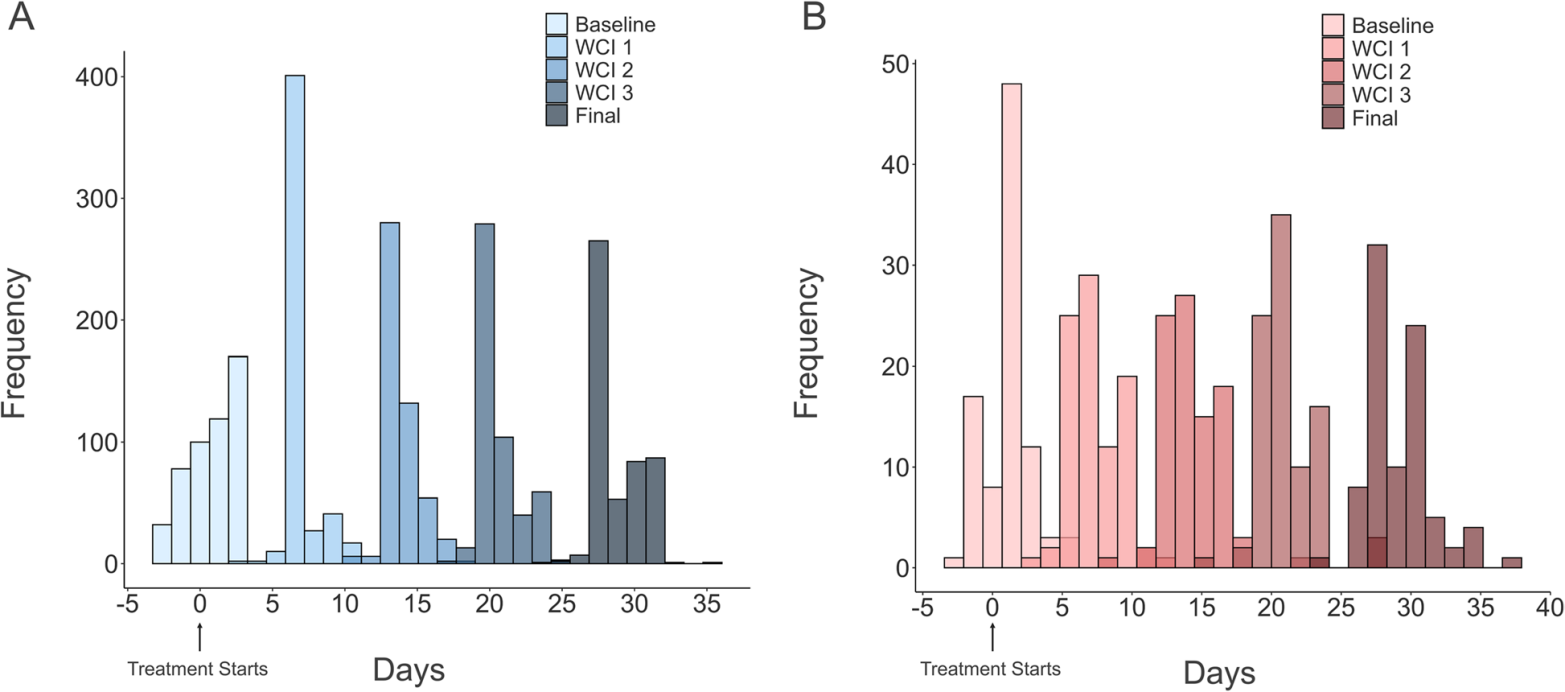
Distributions of overlapping completions dates of each study section for (**A**) the iCBT arm and (**B**) the antidepressant arm. Day ‘0’ depicts treatment start date

On average, participants in both arms completed the baseline assessment ~ 1 day after initiating treatment (Antidepressant: Mean = 1.24, Median = 1, SD = 1.64, range = -3 to + 5 days; iCBT: Mean = 0.77, Median = 1, SD = 1.45, range = -2 to + 4 days). For the iCBT cohort, we had the benefit of some objective data from the iCBT provider to complement the self-report. The median difference between self-reported treatment start date and the day participants first registered on the iCBT platform was + 1 day (Mean = 1.76, SD = 2.90, range = -1 to + 20) (i.e., they registered for iCBT on the platform 1 day before they reported to us they would start treatment). The due date for all subsequent assessments were based on the self-report treatment start date, regardless of whether their last assessment was completed slightly early or late. Weekly check-in assessments and the final assessment were provided to participants 1 day before they were due with the instruction to complete them on the following day. Despite this instruction, we found that many participants completed them immediately upon receipt (i.e., 1 day before due date). From the treatment initiation date, weekly check-in 1 was completed on average on day 6 (Mean = 6.78, SD = 1.33), but there were a handful of longer intervals (range = 3–15), weekly check-in 2 was completed on average on day 13 (Mean = 13.88, SD = 1.40, range = 8–22), and weekly check-in 3 was completed on average on day 20 (Mean = 20.81, SD = 1.35, range = 15–28). The final assessment, which was more time-consuming than the weekly check-ins, was completed on average on day 28 (Mean = 28.76, SD = 1.74, range = 23–37). The median interval between treatment initiation and final assessment was 28 days (Mean = 28.70, SD = 1.59, range = 24–36) in the iCBT arm and 29 days (Mean = 29.15, SD = 2.42, range = 23–37) in the antidepressant arm (Fig. 6A, B, and Figure S2).

Participants were requested to complete the baseline and final assessments in a single sitting, taking short breaks between sections. However, for a variety of practical and technical reasons, some participants were only able to partially complete their baseline or final assessment before returning later to complete the remaining sections. We defined participants as completing the section in 1 sitting if they did not take a break exceeding 4 hours between any of the study sections. Using this cut-off, 9% (*N* = 47) of participants in the iCBT arm and 23% of participants (*N* = 21) for the antidepressant arm did not complete the baseline assessment in a single sitting. The trend is similar for the final session, 9% (*N* = 43) in the iCBT arm and 16% (*N* = 15) in the antidepressant arm did not complete it in a single sitting. Of those who completed their assessments in a single sitting, the median time it took participants to complete the baseline assessment was 1.63 hours (SD = 0.77) and the median time to complete the final assessment was 1.30 hours (SD = 0.85). Participants were more likely to complete the brief weekly check-ins during daytime (6am-6 pm: 76%) when compared to the baseline and final assessments (both 59%, *X*^2^ = 62.71, *p* < 0.001).

### Data Quality

**Fig. 7:**
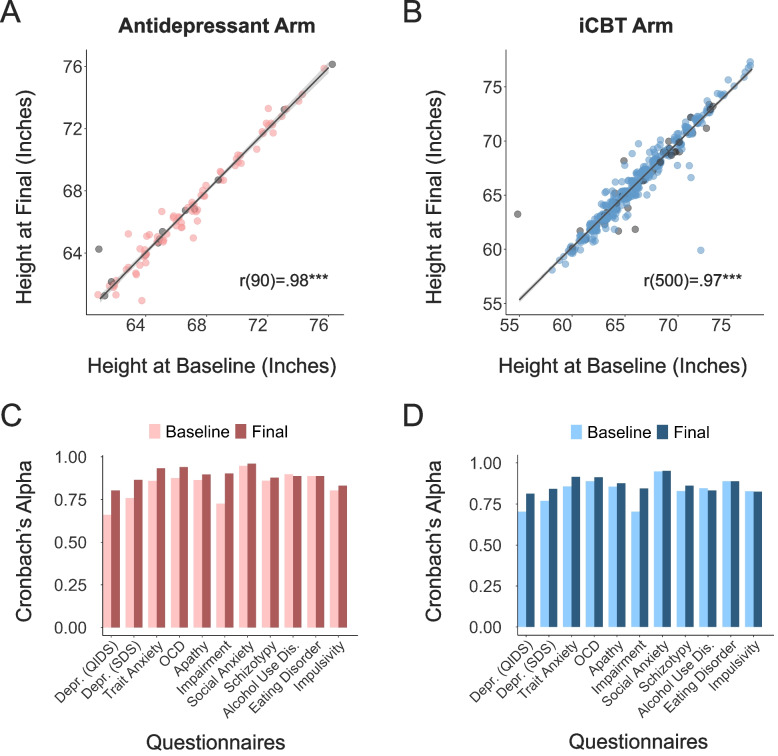
Data Quality indicators. Correlation of height (in inches) was gathered at the baseline and final assessments in (**A**) the Antidepressant arm, *r* = 0.98 and (**B**) the iCBT arm, *r* = 0.97. Participants who failed at least 1 attention check are coloured grey. Internal consistency of the self-report questionnaires (Cronbach’s alpha) for the (**C**) Antidepressant arm and the (**D**) iCBT arm

At baseline, 66% reported being distracted in some way (iCBT: *n* = 310, 65%; Antidepressant: *n* = 61, 75%). Overall, the most common types of distractions endorsed were family and friends (37%), background noise (32%), and phone (28%). In relation to intoxicating substances, at baseline, just 3% of participants informed us that they had taken 1 of our defined substances within 5 hours of starting the study (iCBT: *n* = 14, 3%; Antidepressant: *n* = 4, 5%). Of the very few participants who reported any form of substance use, 13 had consumed alcohol (2%), 5 reported marijuana use (< 1%) and 2 people reported using opiates (< 1%) (see Table S7 and S8 in Additional File 1 – Supplementary Materials for similar trends in distraction and substance use items at the final assessment).^4^ In terms of our inattention ‘catch questions’, 11% (*n* = 63) of participants failed at least 1 of the 6 attention checks embedded in the study (iCBT: *n* = 51, 10%; Antidepressant: *n* = 12, 13%). The majority of inattentive participants were only inattentive at one time (*n* = 47, 8% of total sample), with just 3% of the total sample (*n* = 16) failing more than 1 attention check. People were more likely to be inattentive at certain timepoints in the study, *X*^2^ = 25.32 (5), *p* < 0.001. The longer, more burdensome assessment sessions had more attention lapses (baseline: check 1 *n* = 17, check 2 *n* = 35; final: check 1 *n *= 21) than the 3 brief weekly check-ins (1 check at each: *n* = 12, *n* = 11, *n* = 10, respectively). To further assess data quality, we examined participants’ consistency in reporting their height at the baseline and final assessments. Height reports were reliably measured across the two time points (ICC1 = 0.97), and there was no significant difference in the absolute size of the discrepancy of height reports based on whether participants were classed as inattentive or not, t(67) = -1.66, *p* = 0.10 (Fig. 7A, B). Finally, we examined the internal consistency of self-report symptom assessments. At baseline, Cronbach’s alpha was good for all scales (i.e., r > 0.7), ranging from 0.71–0.95, and this rose to 0.81–0.95 at the final assessment (Fig. 7C, D). Full results are presented in Table S9 in Additional File 1 – Supplementary Materials.

### Qualitative Feedback

Of 155 invited, 135 participants completed the online feedback survey from 19^th^ June 2020 to 13^th^ October 2020, giving a response rate of 87%. Data were analysed for 4 open-ended free-text questions concerning what participants liked and disliked about the study, what they suggested could be added to the study, and whether they found the payment schedule to be satisfactory (see section 'Qualitative Data Analysis' in Additional File 1 – Supplementary Materials). When asked *“What did you like about the study?”*, the most prominent theme emerging from the responses relates to *Self-Reflection* (40%). Participants liked how the study prompted them to reflect on aspects of their mental health they would not have done otherwise and helped them keep track of treatment progress through the weekly check-ins. Another major theme was that participants found the study *Easy to Complete* (33%), citing convenience in terms of both online accessibility and flexibility with respect to assessment completion times and dates, the inclusion of breaks and email reminders, and the clarity of instructions. A proportion of respondents said they liked the *Gamified Tasks* (24%) which some found interactive and challenging, and a further 20% of participants reported feeling aligned in general with the *Study’s Mission*. Only 4% of participants reported *Payment* as what they liked about the study. Although 24% of participants reportedly liked the games, when asked *“What did you dislike about the study?”*, a large number (89%) also cited the *Gamified Tasks* (89%), which were felt to be tedious (e.g., “repetitive”, “boring”, “lengthy”), and in some cases confusing, frustrating, and too difficult. The second most prominent theme in response to this question about dislikes referred to the overall *Study Design and Mechanics* (13%). Some did not like the length of baseline and follow-up assessments and overall time-commitment involved, while others had problems with study coordination, administrative or other logistical problems.

In terms of suggestions for additions to the study in future, most of the respondents suggested additional aspects of *Self-Report* (83%). Recommendations ranged from the inclusion of free-text and experience sampling to including a broader range of questions on treatment information, psychological states and behaviours, demographics, lifestyle, physical health, environmental factors, own perceptions of change/symptoms/problems, and positive mood. Another area for improvement pertained to the *Study Mechanics and Design*, including extending the overall study duration and including a longer-term follow-up assessment. A good proportion of participants (30%) reported there was *Nothing* they wished to add to the study. In relation to the payment schedule, participants were overwhelmingly satisfied, with only 7% citing a negative experience (e.g., missed/delayed payments, not worth the time-commitment). Most participants (71%) rated the payment schedule positive or very positive and some (33%) were neutral about it (e.g., “fine”, “no complaints”, “appropriate”).

## Discussion

The adoption of online data collection in psychiatric research has seen a dramatic increase in recent years [59], but much of this research remains cross-sectional and correlational in nature. The Precision in Psychiatry (PIP) study extends this conventional approach to create a foundation for longitudinal treatment prediction research in psychiatry. We recruited and screened a large sample of individuals receiving iCBT (baseline *N* = 600, final *N* = 502) and a smaller sample receiving antidepressant medication (baseline *N* = 110, final *N *= 92). For eligible patients, we acquired an extensive range of self-report and cognitive measures (> 600 variables) at baseline and after 4 weeks of treatment, in addition to brief weekly check-ins. In what follows, we discuss the benefits and limitations of this approach and put forward recommendations for future studies (Table 4).

**Table 4:** Practical Guidance for Internet-Based Treatment Prediction Research

Practical Guidance for Internet-Based Treatment Prediction Research
1.**Keep Assessments Brief** Retention was high for brief, self-report assessments and in particular weekly check-ins were well-received by patients wishing to track their progress through treatment. Cognitive tests were by far the most disliked component of our study. Considerable work is needed to make these more tolerable for participants
2.**Ensure Incentives are Aligned** The key to quality data in an online environment is to keep incentives aligned. Participants in our study resonated with the mission of the study and/or enjoyed the opportunity for self-reflection. Future research should be sensitive to these motivations and (i) communicate the mission of the study clearly, early, and often, (ii) supply participants with information about study outcomes at the time of publication, (iii) solicit feedback from participants and (iv) consider a graphical display where service users can visualise their progress throughout treatment
3.**Make Participation Easy** The ease of participation is imperative to achieving successful online recruitment, for example, allowing participants to complete assessments remotely and at a time convenient to them. In addition to a PC/laptop, smartphone and smartwatch may be incorporated in future for increased convenience in online data collection. They can further facilitate the collection of different sorts of data, such as mobility data, sleep, and experience sampling data
4.**Issue Regular Reminders, be Flexible and Pragmatic** To encourage retention, a timely reminder for each assessment should be delivered a day prior to due date, and a small window for completion may be provided to increase flexibility for participants to complete each assessment. Sensitivity analyses can be used to ensure late or early assessments do not confound results
5.**Data Quality is not a Given** Data quality indicators (e.g., catch questions, distraction probes, and stable variables for high test–retest reliability analysis) should continue to be included for assessing the quality of self-report online data. The online research environment changes and is potentially vulnerable to bots or dishonest respondents. To reduce the threat this poses to valid research, recruitment should be targeted to those initiating treatment and include a validation check (prescription photo, iCBT registration)

### Recruitment at Scale, at Speed

The major success of the study is that it enabled us to recruit a large cohort of patients undertaking treatment for depression in a relatively short period of time. This was most evident in the iCBT arm, where we reached a maximum recruitment rate of 59 patients completing the baseline assessment per month. This corresponded to just over 500 full study completers within one year and a half. While the antidepressant arm was slower and more expensive to recruit for, we nonetheless gathered data from close to 100 individuals in 13 months, which compared to conventional strategies for recruiting participants with clinical diagnoses, was rapid. This approach also benefitted from high retention rate, where 93% of participants were retained for 3 weeks, and that dropped to 84% at week 4, following the final (lengthier) assessment (1.5 hours). If future research does not require detailed cognitive and clinical follow-up data (i.e., studies focused purely on prediction), this suggests one can expect retention > 90%, if follow-up assessments are brief (e.g., restricting to 1 or 2 self-report outcome measures). Qualitative feedback from users suggests that the flexibility of the study design may have helped us to recruit and retain participants. When participants are able to take part from any location, and at any time, this reduces logistic challenges associated with traditional, in-person data collection methods (e.g., travel to in-person locations, 9–5 participation hours) and makes research available to people often underrepresented in research (e.g., those from rural areas, socially anxious, more severely disabled). Treatment adherence for those who remained in the study was very high at >  = 97%.

### Compatibility with Digital Therapy

Digital psychological interventions such as iCBT are becoming increasingly popular as they allow greater access to care at a reduced cost, while demonstrating similar effectiveness for those requiring low intensity intervention [1, 60, 61]. There exists, however, little basic research examining the mechanisms of therapeutic change in iCBT, how it affects cognition, brain function, or indeed, who it is best suited to and why. We see this as an important opportunity for future work for several reasons. iCBT lends itself well to systematic research as the therapeutic content that patients have access to is standardised and reproducible, which solves issues of both inter-clinician and intra-clinician variability in the delivery of in-person CBT and leads to more generalisable insights. Individual variability in engagement with the online platform can be tracked precisely via granular and objective treatment data (e.g., what modules, when, and for how long), which can be mined to understand moderators of treatment success [62, 63]. This may be particularly useful for researchers and clinical providers aiming to identify active ingredients of successful CBT, for personalisation, precision and more. This combination of digitised therapy and digitised research may thus provide a much more direct route to real-world clinical integration than other less integrated approaches.

### Non-Random Assignment in Naturalistic Design

The observational nature of this study reflects the ‘real world’ of treatment allocation (i.e., non-randomised), which places a fundamental limit on causal inference. Though it does not solve the problem of non-random assignment, we included more than one observational arm, which allows us to assess the generalisability and specificity of any treatment prediction model we develop to new cohorts and treatments. In terms of demographics, participants in both iCBT and antidepressant arms were primarily white, female, employed, third level educated, which limits the generalisability of these findings. These sample characteristics are comparable to other large-scale studies recruiting for antidepressant treatment [2] and iCBT [55], indicating that this lack of generalisability is not a problem unique to digital treatment research, but something that all research in this area needs to work to address. Participants in the antidepressant arm were marginally younger and more likely to be unemployed, but most notably they had more severe clinical presentations and symptoms than their iCBT counterparts. This was expected as it follows the guidelines of the Improving Access to Psychological Therapies (IAPT) program in NHS, where iCBT is typically prescribed first for mild to moderate depression before antidepressant medication is considered [64]. Given this, the finding that by week 4, participants in the antidepressant arm experienced greater symptom reduction, should be interpreted with caution. Prior research suggests comparable effectiveness of the two treatments [65] and the short timeframe of our study and the self-paced nature of the iCBT intervention may have made for a weaker overall ‘dose’ for this arm. Some participants were receiving concurrent, overlapping treatments (8% of patients in the iCBT arm taking medication, and 36% of the antidepressant arm receiving some form of psychotherapy). This is a significant limitation of the inclusive study design we adopted, and insights regarding specificity or generality of any effects should be supported with sensitivity analyses (i.e., excluding participants undertaking concurrent treatments).

### Lack of Experimental Control

While we could not control when and if participants would complete each study section as per their schedule, to make participation as convenient and flexible as possible, we issued each study section one day before due-date and allowed participants a 4-day window to complete it. As a result, participants on average completed assessments 1 day earlier than they were due, and participants overall differed in the intervals between starting treatment and completing baseline and subsequent sessions. While these differences were minimised, issues of timing are some of the most challenging for researchers working with internet-based methods to manage. As previously mentioned, online studies primarily rely on self-report, rather than clinician-assigned diagnoses or severity assessments. This raises legitimate concerns regarding the reliability and validity of online data gathered in a less-controlled environment when compared to traditional, in-lab/in-clinic settings. Online studies can be susceptible to inattentive and careless responding, as is the case for other forms of online research (e.g., crowdsourcing) [66]. At a minimum, prior research suggests that individuals tend to follow task instructions better when tested in-person versus at home [67]. Our analyses of the quality of the baseline data we gathered revealed that some participants failed to complete their assessments in a single sitting (11%), most reported being distracted during the study session (63%), and a small few had even consumed intoxicating substances (3%). On the extreme end, some online platforms (e.g. Amazon’s Mechanical Turk) are suffering from major quality control issues, with a recent paper finding that > 50% of respondents reported their own gender identity inconsistently at two time-points [68]. We believe these more serious risks can be mitigated by adopting a more targeted recruitment protocol such as that described here, i.e., advertising the study to only those individuals eligible, using validation steps (prescription upload, registration requirement for iCBT), and ensuring that the mission of the study is aligned to the goals of the participants (i.e., improving mental health treatment) [68]. In terms of our more objective quality checks, just 11% of subjects were caught by any of our attention checks when filling in online questionnaires. Overall, we found the data to be of excellent quality; height (in inches) self-reported at week 0 and week 4 had near perfect inter-rater reliability. This exceeds the 2-week test–retest reliability of height (in adolescents) gathered using paper booklets (*r* = 0.93) [69]. Internal consistency of the self-report questionnaires administered was also high. QIDS had the lowest Cronbach’s alpha at baseline of 0.71, comparable to that observed in another patient sample at treatment outset (alpha = 0.72) [24]. In terms of measurement properties, inter-rater reliability (clinician agreement) for some of the most common mental health conditions, including depression, is relatively low for the criteria of the Diagnostic and Statistical Manual of Mental Disorders-5 (DSM-5) [70], while self-report assessments enjoy much higher reliability, both in-person [71] and online [72]. Although self-report has these advantages, it may be less valid for use in mental health populations where insight is compromised.

## Conclusion

Depression is a highly heterogeneous disorder for which no single treatment intervention is universally effective. We need to move from a trial-and-error approach to treatment to one that is precise and where possible personalised. To this end, researchers are currently exploring the potential of developing clinical decision tools by training machine learning algorithms to predict clinical outcomes. In order to obtain robust predictions, we need substantially larger sample sizes than is typical in the field. Our data suggest that Internet-based methods can achieve this, allowing us to gather rich, complex datasets from large cohorts, with measurable indicators of treatment adherence and engagement. We hope that the detailed data we have provided in this paper provides a working template for future Internet-based treatment studies in psychiatry.

## Supplementary Information

**Additional file 1.** 

**Additional file 2.**

## Data Availability

The datasets used and/or analysed during the current study are available from the corresponding author on reasonable request. All data from the PIP study will be made publicly available once the primary treatment prediction machine learning analysis of the project are published on a pre-print server.

## Abbreviations

AES: Apathy Evaluation Scale
ANOVA: Analysis of variance
AUDIT: Alcohol Use Disorder Identification Test
BIS: Barratt Impulsivity Scale
CBT: Cognitive Behavioural Therapy
CTQ: Childhood Trauma Questionnaire
CIRS: Cumulative Illness Rating Scale
DSM-5: Diagnostic and Statistical Manual of Mental Disorders-5
EAT-26: Eating Attitude Test-26
EMBARC: Establishing Moderators and Biosignatures of Antidepressant Response for Clinical Care for Depression
GAD: Generalised Anxiety Disorder
iCBT: Internet-based cognitive behavioural therapy
iSPOT-D: International Study to Predict Optimised Treatment—in Depression
IAPT: Improving Access to Psychological Therapies
LSAS: Liebowitz Social Anxiety Scale
MRI: Magnetic resonance imaging
MSPSS: Multidimensional Scale of Perceived Social Support
NHS: National Health Service
OCD: Obsessive-compulsive disorders
OCI-R: Obsessive-Compulsive Inventory – Revised
PHQ-15: Patient Health Questionnaire-15
PIP: Precision in Psychiatry study
PReDICT: Predictors of Remission in Depression to Individual and Combined Treatments
PSS: Perceived Stress Scale
QIDS-SR: Quick Inventory of Depressive Symptomatology – Self-Report
SDS: Self-Rating Depression Scale
SNRI: Serotonin-norepinephrine reuptake inhibitors SRRS: Social Readjustment Rating Scale
SSMS: Short Scales for Measuring Schizotypy
SSRI: Selective serotonin reuptake inhibitors
STAI: State-Trait Anxiety Inventory
STAR*D: Sequenced Treatment Alternatives to Relieve Depression
TCA: Tricyclic antidepressants
WCI: Weekly check-in
WSAS: Work and Social Adjustment Scale

## Footnotes

1 The first informal participant of the antidepressant arm was recruited in December 2018, however, as this was during the pilot recruitment phase, this participant and their recruitment date were therefore not considered in the study.

2 N = 2 in the iCBT arm identified as outliers were removed for this analysis (i.e., they have QIDS % changes larger than ± 3 standard deviations from mean).

3 N = 4 in the iCBT arm and N = 1 in the antidepressant arm identified as outliers were removed for this analysis (i.e., they have WSAS % changes larger than ± 3 standard deviations from mean).

4 At baseline, N = 33 were missing data for distraction and substance use data quality item checks.
