# Supplementary material for: The Precision in Psychiatry (PIP) study: Testing an internet-based methodology for accelerating research in treatment prediction and personalisation
Source: BMC Psychiatry. 2023 Jan 11;23:25. doi: 10.1186/s12888-022-04462-5 (PMC9832676; doi:10.1186/s12888-022-04462-5)
Supplement: Supplementary file 1 — Additional file 1. [file 12888_2022_4462_MOESM1_ESM.docx]

Additional File 1.

PIP Supplementary Material

Table of Contents

[Schedule of Assessments 2](#_Toc115856984)

[Table S1. Measures collected per study stage, from screening, baseline, weekly check-ins to the final assessment. 2](#_Toc115856985)

[Descriptives of Medication in the Antidepressant Arm 4](#_Toc115856986)

[Table S2. Proportion of participants receiving medication from the different drug classes (N = 92). 4](#_Toc115856987)

[Table S3. List of antidepressant medications prescribed (N = 92). 4](#_Toc115856988)

[Table S4. List of other non-antidepressant medication prescribed (N = 92). 4](#_Toc115856989)

[Baseline Characteristics of Baseline Completers 5](#_Toc115856990)

[Table S5. Baseline demographics of baseline completers (N = 710). 5](#_Toc115856991)

[Table S6. Baseline clinical characteristics of baseline completers (N = 710). 6](#_Toc115856992)

[Baseline Clinical Score Distribution 8](#_Toc115856993)

[Figure S1. Baseline clinical symptom total score distribution for the iCBT and antidepressant arm. 8](#_Toc115856994)

[Study Schedule Compliance 9](#_Toc115856995)

[Figure S2. Distributions of overlapping completions dates of baseline, weekly, and final assessments. 9](#_Toc115856996)

[Reliability and Validity of Self-Report Data 10](#_Toc115856997)

[Table S7. Data quality checks on completers (N = 573) at the final assessment. 10](#_Toc115856998)

[Table S8. Distraction types and substance uses of distracted and/or intoxicated completers at the final assessment. 10](#_Toc115856999)

[Table S9. Internal consistency of self-report scales at baseline and final assessments (Cronbach’s alpha). 11](#_Toc115857000)

[Between-Group Comparisons 12](#_Toc115857001)

[iCBT Program Effects 15](#_Toc115857002)

[Table S10. Summary statistics of iCBT treatment engagement over 4 weeks (N = 491). 15](#_Toc115857003)

[Figure S3. iCBT treatment engagement data. 17](#_Toc115857004)

[Sensitivity Analyses 18](#_Toc115857005)

[Excluding inattentive responders on clinical change. 18](#_Toc115857006)

[Comparison of baseline QIDS-SR symptomatology between dropouts and completers. 19](#_Toc115857007)

[Inattentive responders on response consistency indicators. 19](#_Toc115857008)

[Qualitative Data Analysis 20](#_Toc115857009)

[References 21](#_Toc115857010)

# Schedule of Assessments

## Table S1. Measures collected per study stage, from screening, baseline, weekly check-ins to the final assessment.

|  | Schedule of Assessments | | | | | | | |
| --- | --- | --- | --- | --- | --- | --- | --- | --- |
| Measure | Screening | Baseline | WCI 1 | | WCI 2 | | WCI 3 | Final |
| Socio-Demographics |  |  |  |  | |  | |  |
| Age | X |  |  |  | |  | |  |
| Sex |  | X |  |  | |  | |  |
| Country of Residence |  | X |  |  | |  | |  |
| Marital Status |  | X |  |  | |  | |  |
| Education Level |  | X |  |  | |  | |  |
| Employment Status |  | X |  |  | |  | |  |
| Subjective Social Status |  | X |  |  | |  | |  |
| Physical Health and Lifestyle |  |  |  |  | |  | |  |
| Exercise |  | X |  |  | |  | |  |
| Diet |  | X |  |  | |  | |  |
| Drug Use |  | X |  |  | |  | |  |
| Physical Health Comorbidities (CIRS) |  | X |  |  | |  | |  |
| Pain (PHQ-15) |  | X |  |  | |  | |  |
| Smoking |  | X |  |  | |  | | X |
| Height & Weight |  | X |  |  | |  | | X |
| Psychosocial |  |  |  |  | |  | |  |
| Stressful Life Events (SRRS) |  | X |  |  | |  | |  |
| Childhood Trauma (CTQ) |  | X |  |  | |  | |  |
| Perceived Social Support (MSPSS) |  | X |  |  | |  | |  |
| Perceived Stress (PSS) |  | X |  |  | |  | | X |
| Clinical |  |  |  |  | |  | |  |
| Chronicity |  | X |  |  | |  | |  |
| Psychiatric Diagnoses (Self) |  | X |  |  | |  | |  |
| Psychiatric Diagnoses (Family) |  | X |  |  | |  | |  |
| Miscellaneous Psychiatric Symptoms |  | X |  |  | |  | |  |
| Apathy (AES) |  | X |  |  | |  | | X |
| Alcoholism (AUDIT) |  | X |  |  | |  | | X |
| Impulsivity (BIS) |  | X |  |  | |  | | X |
| Eating Disorder (EAT-26) |  | X |  |  | |  | | X |
| Social Anxiety (LSAS) |  | X |  |  | |  | | X |
| Schizotypy (SSMS) |  | X |  |  | |  | | X |
| Depression (SDS) |  | X |  |  | |  | | X |
| State Anxiety (STAI-T) |  | X |  |  | |  | | X |
| Obsessive Compulsive Disorder (OCI-R) |  | X | X | X | | X | | X |
| Depression (QIDS-SR) |  | X | X | X | | X | | X |
| Functional Impairment (WSAS) | X |  | X | X | | X | | X |
| Treatment |  |  |  |  | |  | |  |
| Antidepressant Medication Type^a^ | X |  |  |  | |  | |  |
| Treatment History |  | X |  |  | |  | |  |
| Treatment Expectation |  | X |  |  | |  | |  |
| Treatment Adherence |  |  | X | X | | X | |  |
| Antidepressant Medication Side Effects^a^ |  |  | X | X | | X | |  |
| Antidepressant Medication Dosage^a^ |  |  | X | X | | X | |  |
| Extra Treatment Information |  |  | X | X | | X | |  |
| Concurrent Medication Treatment | X |  | X | X | | X | |  |
| Concurrent Psychotherapy Treatment |  | X | X | X | | X | |  |
| iCBT Treatment Engagement^b^ |  | X | X | X | | X | | X |
| Cognitive Performance |  |  |  |  | |  | |  |
| Perceptual Decision-Making Task |  | X |  |  | |  | | X |
| Two-Step Reinforcement-Learning Task |  | X |  |  | |  | | X |
| Learning Under Volatility Task |  | X |  |  | |  | | X |
| Abstract Reasoning Test |  | X |  |  | |  | | X |
| Data Quality Probes |  |  |  |  | |  | |  |
| Distraction Probe |  | X |  |  | |  | | X |
| Substance Use Probe |  | X |  |  | |  | | X |
| Attention Check OCI |  | X |  |  | |  | |  |
| Attention Check WSAS | X |  | X | X | | X | | X |

^a^Only collected for the antidepressant arm

^b^Only collected for the iCBT arm

# Descriptives of Medication in the Antidepressant Arm

## Table S2. Proportion of participants receiving medication from the different drug classes (N = 92).

| Drug Class | Frequency (N) | Percent (%) |
| --- | --- | --- |
| Selective serotonin reuptake inhibitor (SSRI) | 79 | 85.87 |
| Serotonin–norepinephrine reuptake inhibitor (SNRI) | 12 | 13.04 |
| Atypical antidepressant | 6 | 6.52 |
| Tricyclic antidepressant (TCAs) | 2 | 2.17 |
| Other non-antidepressant | 5 | 5.43 |

The total percentage exceeds 100 as participants can be taking more than one medication of different classes.

## Table S3. List of antidepressant medications prescribed (N = 92).

| Name | Frequency (N) | Percent (%) | Mean Dose | SD Dose | Range Dose |
| --- | --- | --- | --- | --- | --- |
| SSRI |  |  |  |  |  |
| Sertraline | 37 | 40.22 | 60.20 | 29.59 | 2.5-150 |
| Escitalopram | 17 | 18.48 | 12.65 | 5.34 | 5-20 |
| Fluoxetine | 14 | 15.22 | 22.86 | 7.26 | 20-40 |
| Citalopram | 7 | 7.61 | 20 | 5.78 | 10-30 |
| Paroxetine | 2 | 2.17 | 20 | 0 | NA |
| Fluvoxamine | 1 | 1.09 | 20 | 0 | NA |
| Vortioxetine | 1 | 1.09 | 10 | 0 | NA |
| SNRI |  |  |  |  |  |
| Venlafaxine | 8 | 8.70 | 51.56 | 27.90 | 37.5-112.5 |
| Duloxetine | 3 | 3.26 | 50 | 17.32 | 30-60 |
| Levomilnacipran | 1 | 1.09 | 20 | 0 | NA |
| Tricyclic |  |  |  |  |  |
| Amitriptyline | 1 | 1.09 | 10 | 0 | NA |
| Dosulepin | 1 | 1.09 | 75 | 0 | NA |
| Atypical |  |  |  |  |  |
| Mirtazapine | 6 | 6.52 | 23.33 | 12.11 | 15-45 |

All participants were taking at least one medication from the list above. Additional medications were listed in Table S3.

## Table S4. List of other non-antidepressant medication prescribed (N = 92).

| Name | Frequency (N) | Percent (%) | Mean Dose | SD Dose | Range Dose |
| --- | --- | --- | --- | --- | --- |
| Propranolol^a^ | 1 | 1.09 | NA | NA | NA |
| Olanzapine^a^ | 1 | 1.09 | NA | NA | NA |
| Zolpidem | 1 | 1.09 | 10 | 0 | NA |
| Sumatriptan | 1 | 1.09 | 50 | 0 | NA |
| Lamotrigine | 1 | 1.09 | 150 | 0 | NA |

In addition to their antidepressant medications, participants reported other medication they were taking for their brain health, entered in free-response boxes. ^a^Missing N=1 dose

# Baseline Characteristics of Baseline Completers

## Table S5. Baseline demographics of baseline completers (N = 710).

| Sample Characteristics | iCBT | | |  | Antidepressant | | | t / X^2^ (df) | p |
| --- | --- | --- | --- | --- | --- | --- | --- | --- | --- |
|  | N | % | Median (SD) |  | N | % | Median (SD) |  |  |
|  |  |  |  |  |  |  |  |  |  |
| Sex | 600 |  |  |  | 110 |  |  | 2.58 (3) | 0.46 |
| Female | 466 | 77.67 |  |  | 79 | 71.82 |  |  |  |
| Male | 128 | 21.33 |  |  | 29 | 26.36 |  |  |  |
| Other | 6 | 1.00 |  |  | 2 | 1.82 |  |  |  |
|  |  |  |  |  |  |  |  |  |  |
| Country | 596 |  |  |  | 110 |  |  | 132.71 (2) | < 0.001 |
| UK | 487 | 81.71 |  |  | 40 | 36.36 |  |  |  |
| Ireland | 100 | 16.78 |  |  | 47 | 42.73 |  |  |  |
| Other | 9 | 1.51 |  |  | 23 | 20.91 |  |  |  |
|  |  |  |  |  |  |  |  |  |  |
| Age | 598 |  | 29 (11.00) |  | 109 |  | 26 (9.73) | -2.22 (705) | 0.03 |
|  |  |  |  |  |  |  |  |  |  |
| Marital Status | 600 |  |  |  | 110 |  |  | 1.86 (5) | 0.87 |
| Single | 239 | 39.83 |  |  | 47 | 42.73 |  |  |  |
| In a Relationship | 178 | 29.67 |  |  | 36 | 32.73 |  |  |  |
| Married | 145 | 24.17 |  |  | 21 | 19.09 |  |  |  |
| Divorced | 21 | 3.50 |  |  | 3 | 2.73 |  |  |  |
| Separated | 16 | 2.67 |  |  | 3 | 2.73 |  |  |  |
| Widowed | 1 | 0.17 |  |  | 0 | 0.00 |  |  |  |
|  |  |  |  |  |  |  |  |  |  |
| Education Level | 600 |  |  |  | 110 |  |  | 4.47 (2) | 0.11 |
| <Third Level | 142 | 23.67 |  |  | 16 | 14.55 |  |  |  |
| Some/Complete  Third Level | 323 | 53.83 |  |  | 66 | 60.00 |  |  |  |
| >Third Level | 135 | 22.50 |  |  | 28 | 25.45 |  |  |  |
|  |  |  |  |  |  |  |  |  |  |
| Employment Status | 502 |  |  |  | 110 |  |  | 10.16 (2) | 0.006 |
| Employed | 414 | 69.00 |  |  | 59 | 53.64 |  |  |  |
| Unemployed | 180 | 30.00 |  |  | 50 | 45.45 |  |  |  |
| Retired | 6 | 1.00 |  |  | 1.0 | 0.91 |  |  |  |
|  |  |  |  |  |  |  |  |  |  |
| Subjective Social Status | 600 |  | 4 (1.69) |  | 110 |  | 4 (1.96) | 1.44 (708) | 0.15 |
|  |  |  |  |  |  |  |  |  |  |

Outliers were not excluded in the descriptive analyses of demographic characteristics.

## Table S6. Baseline clinical characteristics of baseline completers (N = 710).

| Sample Characteristics | iCBT | | |  | Antidepressant | | | | t / X^2^ (df) | p |
| --- | --- | --- | --- | --- | --- | --- | --- | --- | --- | --- |
|  | N | % | Median (SD) |  | N | % | Median (SD) | |  |  |
|  |  |  |  |  |  |  | |  |  |  |
| No. of Current Diagnosis | 600 |  |  |  | 110 |  | |  | 31.37 (2) | < 0.001 |
| None | 192 | 32.00 |  |  | 8 | 7.27 | |  |  |  |
| One | 217 | 36.17 |  |  | 45 | 40.91 | |  |  |  |
| >One | 191 | 31.83 |  |  | 57 | 51.82 | |  |  |  |
|  |  |  |  |  |  |  | |  |  |  |
| Types of Diagnoses^a^ | 600 |  |  |  | 110 |  | |  | 9.57 (5) | 0.09 |
| None | 192 | 32.00 |  |  | 8 | 7.27 | |  |  |  |
| Depression | 287 | 47.83 |  |  | 81 | 73.64 | |  |  |  |
| GAD | 245 | 40.83 |  |  | 63 | 57.27 | |  |  |  |
| Panic Disorder | 30 | 5.00 |  |  | 5 | 4.55 | |  |  |  |
| PTSD | 25 | 4.17 |  |  | 13 | 11.82 | |  |  |  |
| OCD | 25 | 4.17 |  |  | 5 | 4.55 | |  |  |  |
| Others | 46 | 7.67 |  |  | 13 | 11.82 | |  |  |  |
|  |  |  |  |  |  |  | |  |  |  |
| Family with Mental Disorders | 600 |  |  |  | 110 |  | |  | 3.47 (3) | 0.33 |
| None | 251 | 41.83 |  |  | 37 | 33.64 | |  |  |  |
| One | 187 | 31.17 |  |  | 35 | 31.82 | |  |  |  |
| Two | 92 | 15.33 |  |  | 21 | 19.09 | |  |  |  |
| ≥Three | 70 | 11.67 |  |  | 17 | 15.45 | |  |  |  |
|  |  |  |  |  |  |  | |  |  |  |
| No. of Lifetime Episodes | 592 |  |  |  | 109 |  | |  | 7.50 (2) | 0.02 |
| <2 | 63 | 10.64 |  |  | 9 | 8.26 | |  |  |  |
| 2-5 | 292 | 49.32 |  |  | 41 | 37.61 | |  |  |  |
| >5 | 237 | 40.03 |  |  | 59 | 54.13 | |  |  |  |
|  |  |  |  |  |  |  | |  |  |  |
| Age of onset (years) | 588 |  |  |  | 109 |  | |  | 12.20 (2) | 0.002 |
| Childhood (1-12) | 107 | 18.20 |  |  | 31 | 28.44 | |  |  |  |
| Teenage (13-17) | 251 | 42.69 |  |  | 53 | 48.62 | |  |  |  |
| Adulthood (18-70) | 230 | 39.12 |  |  | 25 | 22.94 | |  |  |  |
|  |  |  |  |  |  |  | |  |  |  |
| Current episode length (days) | 543 |  | 187 (2456) |  | 100 |  | | 202 (2320) | 0.01 (641) | 0.99 |
|  |  |  |  |  |  |  | |  |  |  |
| History of Past Treatment | 600 |  |  |  | 110 |  | |  | 9.31 (3) | 0.03 |
| Never Before | 273 | 45.50 |  |  | 36 | 32.14 | |  |  |  |
| Psychotherapy & Medication | 136 | 22.67 |  |  | 32 | 28.57 | |  |  |  |
| Medication only | 95 | 15.83 |  |  | 15 | 13.39 | |  |  |  |
| Psychotherapy only | 96 | 16.00 |  |  | 27 | 24.11 | |  |  |  |
|  |  |  |  |  |  |  | |  |  |  |
| Treatment Expectation (0-9) | 600 |  | 5 (2.05) |  | 110 |  | | 5 (1.91) | -1.23 (708) | 0.22 |
|  |  |  |  |  |  |  | |  |  |  |

^a^Types of Diagnoses: The total number of diagnoses type exceeds the sample size of baseline completers (i.e., participants have the option to pick more than one diagnosis).

Outliers were not excluded in the descriptive analyses of clinical characteristics.

# Baseline Clinical Score Distribution


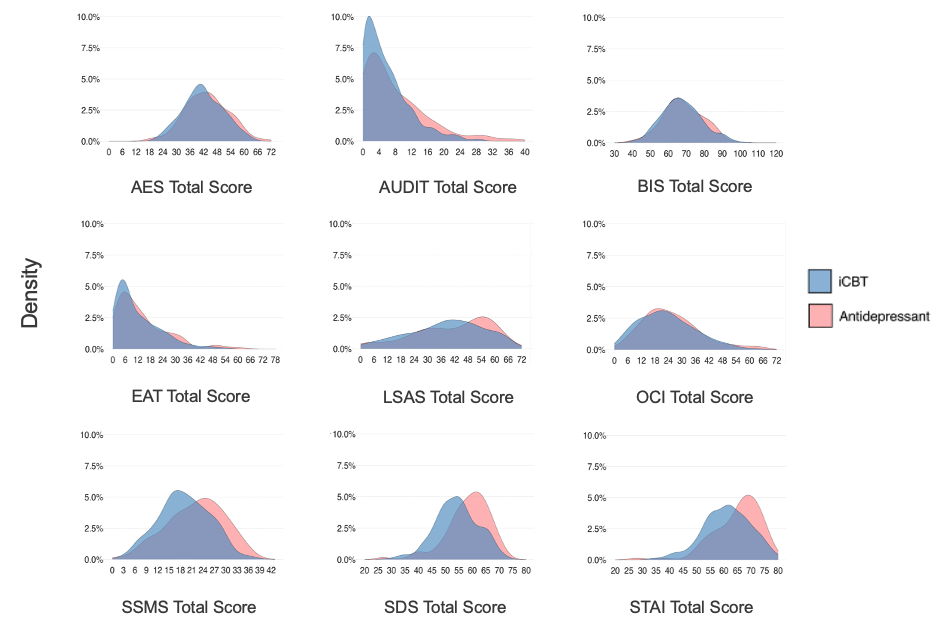


## Figure S1. Baseline clinical symptom total score distribution for the iCBT and antidepressant arm.

# Study Schedule Compliance

##

## Figure S2. Distributions of overlapping completions dates of baseline, weekly, and final assessments.

# Reliability and Validity of Self-Report Data

## Table S7. Data quality checks on completers (N = 573) at the final assessment.

| Data Quality Items | iCBT | |  | Antidepressant | | X^2^ (df) | p |
| --- | --- | --- | --- | --- | --- | --- | --- |
|  | N | % |  | N | % |  |  |
|  |  |  |  |  |  |  |  |
| Distraction | 488 | 100 |  | 85 | 100 | 2.82 (1) | 0.09 |
| Yes | 310 | 63.52 |  | 62 | 72.94 |  |  |
| No | 178 | 36.48 |  | 23 | 27.06 |  |  |
|  |  |  |  |  |  |  |  |
| Substance Use | 488 | 100 |  | 85 | 100 | 0.0005 (1) | 0.98 |
| Yes | 17 | 3.48 |  | 3 | 3.53 |  |  |
| No | 471 | 96.52 |  | 82 | 96.47 |  |  |
|  |  |  |  |  |  |  |  |

At final assessment, N = 21 were missing data for distraction and substance use data quality item checks.

## Table S8. Distraction types and substance uses of distracted and/or intoxicated completers at the final assessment.

| Data Quality Items | N | % |
| --- | --- | --- |
| Distraction Types | 372 |  |
| Background Noise | 125 | 33.60 |
| Family and Friends | 122 | 32.80 |
| Phone | 99 | 26.61 |
| Others | 189 | 50.81 |
|  |  |  |
| Substance Uses | 20 |  |
| Alcohol | 13 | 65.00 |
| Opiates | 4 | 20.00 |
| Marijuana | 3 | 15.00 |
| Others | 1 | 5 |
|  |  |  |

## Table S9. Internal consistency of self-report scales at baseline and final assessments (Cronbach’s alpha).

| Clinical Symptoms | Baseline | |  | Follow-up | | | |
| --- | --- | --- | --- | --- | --- | --- | --- |
|  | α |  |  | α |  | | |
|  |  |  |  |  |  | | |
| Depression (QIDS-SR) | 0.71 |  |  | 0.81 |  | | |
| Impairment (WSAS)^a^ | 0.72 |  |  | 0.86 |  | | |
| Apathy (AES) | 0.86 |  |  | 0.89 |  | | |
| Alcohol Use (AUDIT)^a^ | 0.86 |  |  | 0.85 |  | | |
| Impulsivity (BIS) | 0.82 |  |  | 0.83 |  | | |
| Eating Disorder (EAT) | 0.89 |  |  | 0.89 |  | | |
| Social Anxiety (LSAS) | 0.95 |  |  | 0.95 |  | | |
| OCD (OCI-R) | 0.89 |  |  | 0.92 |  | | |
| Schizotypy (SSMS) | 0.84 |  |  | 0.87 |  | | |
| Depression (SDS) | 0.78 |  |  | 0.84 |  | | |
| Trait Anxiety (STAI) | 0.86 |  |  | 0.92 |  | | |
|  |  |  |  |  | |  |  |

*At baseline, N = 3 were missing AUDIT symptom score and N = 12 were missing WSAS symptom score.

# Between-Group Comparisons

In relation to participant recruitment and attrition, a total sample of N = 594 were recruited for analyses of this study, of which N = 502 (85%) were in the iCBT arm and N = 92 (15%) were in the antidepressant arm. Retention of baseline completers (N = 710) to weekly check-in 3 was excellent for all groups, with no significant differences between iCBT participants (93%) and antidepressant participants (92%), X2 = 0.06 (1), p = 0.80. For the final assessment, those numbers dropped to 84% for both treatment arms.

In relation to patient demographic and clinical characteristics (Table 1 and 2 in the main text), there were no between-group differences in sex, but there was a trend for participants in the iCBT arm to be older than those in the antidepressant arm, t(590) = -1.78, p = 0.08. Participants in the iCBT arm came from the United Kingdom and Ireland (>98%), whereas the antidepressant arm was more international with 17% coming from other regions around the world, X2 = 86.93 (2), p < 0.001. Education level, marital status, and subjective social status did not differ across study arms (all p ≥ 0.16), but there were greater rates of unemployment in the antidepressant arm, X2 = 12.81 (2), p = 0.002. Most participants reported having one or more mental health diagnoses. This differed across arms; 9% of participants in the antidepressant arm had no formal diagnosis versus 31% of the iCBT arm, X2 = 21.67 (2), p < 0.001. This was expected as a proportion of the CBT arm were self-referring. There was no difference across study arms in the number of participants reporting having a family member with a mental health condition, X2 = 1.80 (3), p = 0.62. Participants in the antidepressant arm compared to the iCBT arm reported having more lifetime mental health episodes, X2 = 11.09 (2), p = 0.004, as well as earlier age of onset, X2 = 8.68 (2), p = 0.01, but there were no group differences in self-reported duration of current mental health episode, X2 = 0.05, p = 0.96). There was a trend towards more participants in the iCBT arm being treatment-naïve at 45% versus 32% in the antidepressant arm, X2 = 5.95 (3), p = 0.11. To assess expectations about treatment efficacy, participants rated on a scale from 0-9 (“I don’t expect to feel any better” to “I expect to feel completely better”). Those in the iCBT arm had a trend towards higher expectations (M = 5.02, SD = 2.04) about treatment success than those in the antidepressant arm (M = 4.58, SD = 1.89), t(592) = -1.95, p = 0.05.

In terms of clinical severity at baseline, participants in the antidepressant arm had a mean QIDS-SR score of 16.51 (SD = 4.17), which is conventionally interpreted as corresponding to severe symptoms of depression (24). QIDS-SR scores were somewhat lower in the iCBT arm with a mean of 13.86 (SD = 4.28), which corresponds to moderate depression symptoms (t(592) = 5.47, p < 0.001. For WSAS, those in the antidepressant arm had a mean of 22.73 (SD = 6.84), which indicates severe impairment in functioning. In the iCBT arm, scores were again lower with a mean of 19.02 (SD = 6.65), falling in the moderate range, t(580) = 4.61, p < 0.001. Between-group comparisons of clinical severity of other symptoms assessed at baseline are shown in Table 3 in the main text, where the trend continued for those in the antidepressant arm to have greater severity.

While comparisons between-groups on pre-post 4-week clinical changes are discussed in the main text, here we report between-group differences in relation to clinical change trajectory. To understand the trajectory of treatment response, we examined the weekly depression scores measured on the QIDS-SR. We carried out a linear regression with QIDS-SR as the dependent variable and assessment week (0-4, within-subject) and treatment group (between-subject) as independent variables. As expected, there was a linear effect of assessment week, β = -1.25, p < 0.001, indicating that symptoms decreased with time. There was a main effect of group, such that symptoms were overall lower in the iCBT group (M = 11.9, SD = 4.84) when compared to the antidepressant group (M = 12.9, SD = 5.40), β = -1.86, p < 0.001. There was a significant interaction between group and assessment week, β = 0.44, p < 0.001. Tests of simple effects revealed significant differences across the study arms in depression at baseline only, such that as reported earlier, the iCBT group (M = 13.86, SD = 4.28) initiated the study with lower QIDS scores than the antidepressant group (M = 16.51, SD = 4.17), β = -2.65, p < 0.001. There were however no group differences in depression scores at any other timepoint (all p >= 0.21). To identify where in treatment the biggest gains occurred and if this differed by group, we tested for significant changes in symptoms from week to week. For depression, there were significant reductions in symptoms week-on-week for the iCBT group (all p < 0.001), except for the final interval of week 3 to week 4 (β = -0.1, p = 0.45). Similar trends can be observed for the antidepressant group, where symptoms reduced week-on-week (p <= 0.006) up until the final interval of week 3 to week 4 (β = -0.24, p = 0.50). In terms of between-group differences, participants in the antidepressant arm had larger improvements in depression for the baseline to week 1 interval only compared to iCBT (β = 2.00, p < 0.001), while all other week-to-week changes did not differ across groups (all p > 0.58).

There were also between-group differences in study schedule compliance for the comprehensive assessments at the baseline and final timepoint. Participants in the antidepressant arm completed the baseline assessment 1.24 days after initiating antidepressant medication (Median = 1, SD = 1.64, range = -3 to +5 days). For the iCBT cohort, this was reduced to 0.77 days (Median = 1, SD = 1.45, range = -2 to +4 days), which was significantly shorter than for the antidepressant arm, t(591) = 2.80, p = 0.005. For the iCBT arm, the median interval between treatment initiation and final assessment was 28 days (M = 28.70, SD = 1.59, range = 24-36). In the antidepressant arm, the median interval between treatment initiation and final assessment was 29 days (M = 29.15, SD = 2.42, range = 23-37), which was significantly longer than in the iCBT arm, t(586) = 2.24, p = 0.03. We further compared the two groups in terms of differences in the number of participants who did not complete the baseline and final assessments in one sitting, defined as those who did not take a break exceeding 4 hours between the study sections. At baseline, 9% (N = 47) of participants in the iCBT arm and 23% of participants (N = 21) for the antidepressant arm did not complete the assessment in one single session. At the final assessment, similarly, 9% (N = 43) in the iCBT arm and 16% (N= 15) in the antidepressant arm did not complete it in a single session. Arm differences were significant at baseline, X2 = 13.90 (1), p < 0.001, and at final, X2 = 5.28 (1), p = 0.02.

In relation to group differences in data quality, at baseline, there was a trend for participants in the antidepressant arm (N = 61, 75%) to be more distracted than those in the iCBT arm (N = 310, 65%), X2 = 3.56 (1), p = 0.06. There were no differences between the iCBT and antidepressant study arms on the proportion who took intoxicating substances during participation, X2 = 0.91 (1), p = 0.34 (see Table S7 and S8 for similar trends in distraction and substance use items at final assessment). There were also no significant between-group differences in the proportion of participants who were inattentive (i.e., failed the catch questions) (iCBT N = 51, 10%; Antidepressant N = 12, 13%), X2 = 0.68 (1), p = 0.41.

# iCBT Program Effects

##

Objective treatment engagement data were available from SilverCloud for almost all participants in the iCBT group (N = 491). Consistent with the self-paced nature of iCBT, we found that engagement varied considerably across participants (see Table S10, Figure S3A, and Figure S3B). For example, on average, participants spent just over 2 hours on the platform over the 4-week study period, with values ranging from 2.5 minutes to >17 hours. This corresponded to participants on average viewing 37% of the program they received. On average, participants received two reviews from their supporter and completed 17 activities. Correlational analyses revealed associations between QIDS percent change and total time spent on the platform, r(483) = 0.11, p = 0.01 (Figure S3C), average time per session, r(486) = 0.15, p < 0.001, number of activities, r(486) = 0.11, p = 0.02, and activities per session r(483) = 0.14, p < 0.001.The percentage of the program viewed was not significantly related to percent change, r(486) = 0.07, p = 0.13 (Figure S3D), nor were the number of sessions, the number of reviews by supporters and the number of notes sent from client to supporter (all p > 0.57). Our sample represented users utilising a range of iCBT programs, which include unique tools and modules, but largely overlap in terms of core content. To examine whether the inclusion of a range of programs had effects on treatment response, we compared QIDS percent change across participants undertaking different iCBT program types. Specifically, we compared data from the most utilised programs including Space from Depression, Space from Depression & Anxiety, Space from Anxiety, Space from Generalised Anxiety Disorder (GAD) and Life Skills. Miscellaneous programs were merged into an ‘Others’ category (see Additional File 2 – Variable Directory). A one-way ANOVA demonstrated no significant effect of program type on depression percent change score measured by the QIDS, F(4, 483) = 0.79, p = 0.53, indicating that despite there being some variation in the specific iCBT programs participants received, the treatment effect of iCBT on depression was not dependent on program type (Figure S3E).

## Table S10. Summary statistics of iCBT treatment engagement over 4 weeks (N = 491).

|  | **% (N)** | **Range** |
| --- | --- | --- |
| Program Type |  |  |
| Space from Depression | 24.03 (118) |  |
| Space for Life Skills^a^ | 16.90( 83) |  |
| Space from GAD | 15.27 (75) |  |
| Space from Depression & Anxiety | 14.87 (73) |  |
| Space from Anxiety | 9.57 (47) |  |
| Others | 19.35 (95) |  |
|  | **Median (SD)** |  |
| Total Time Spent (minutes) | 140.62 (162.38) | 2.5-1033.08 |
|  |  |  |
| Number of Sessions^b^ | 11 (11.20) | 2-133 |
|  |  |  |
| Average Time per Session (minutes) | 13.24 (10.75) | 1.16-73.04 |
|  |  |  |
| Number of Activities^c^ | 17 (15.02) | 1-99 |
|  |  |  |
| Activities per Session | 1.50 (0.98) | 0.2-6.71 |
|  |  |  |
| Percentage of Program Viewed | 37.18 (23.54) | 0-100 |
|  |  |  |
| Number of Reviews^d^ | 2 (0.92) | 0-4 |
|  |  |  |
| Number of Review Notes^e^ | 1 (2.47) | 0-42 |
|  |  |  |

^a^All participants recruited through Aware in Ireland was offered Space for Life Skills program. ^b^A single session is defined as each individual instance users logged onto the program. If a specific session has inactivity periods longer than 30 minutes, the next moment of activity will count as a new session. ^c^An activity is defined as an instance a user interacted actively with the platform, e.g., completed a journal entry, used an interactive tool, downloaded, or played relaxation audios. ^d^A review is defined as a message the assigned clinician send to the user so to encourage use of the platform while monitoring and providing feedback about the progress from the last review. ^e^A review note is defined as a reply that the user left for their clinician after a review. Treatment engagement data of the iCBT completers cohort were missing for N = 11 due to technical error.

Figure S3. iCBT treatment engagement data.

(A) Distribution of total time spent (mins) by participants in the iCBT arm across the first 4 weeks of iCBT treatment. (B) Distribution of the proportion of platform viewed by participants in the iCBT arm across the first 4 weeks of iCBT treatment. (C) Relationship between pre-post 4-week depression (QIDS) percent change and total time spent (mins). Correlation analyses showed a significantly positive association between the two variables, r(483) = 0.11, *p* = 0.01. (D) Relationship between pre-post 4-week depression (QIDS) percent change and proportion of platform viewed. Correlation analyses revealed a nominally positive but not statistically significant association between the two variables, r(483) = 0.07, *p* = 0.11. (E) iCBT program specific effects on pre-post 4-week depression (QIDS) percent change. A one-way ANOVA revealed no significant effect of program type on depression percent change score measured by the QIDS, F(5, 482) = 0.75, *p* = 0.59.

# Sensitivity Analyses

##

## Excluding inattentive responders on clinical change.

Pre-Post 4-Week Clinical Changes. For participants in the iCBT arm, depression score significantly decreased by an average of 3.05 points (SD = 4.24) (22%), t(449) = 15.25, p < 0.001, d = 0.72. For participants In the antidepressant arm, depression score significantly decreased by an average of 5.38 points (SD = 4.94) (33%) on the QIDS-SR, t(79) = 9.74, p < 0.001, d = 1.10. A two-way ANOVA confirmed this difference was significant, F(1, 528) = 19.46, p < 0.001. Participants in the antidepressant arm experienced a significantly larger percent reduction in QIDS-SR from baseline than those in the iCBT arm, t(526) = 3.10, p = 0.002, after controlling for baseline severity, t(525) = 2.52, p.adj = 0.01. With regards to response and remission rates, for the iCBT arm, by week 4, 39% of participants have achieved ‘early response’, 17% of participants have achieved ‘response’, and 13% of participants have achieved ‘remission’. Participants in the antidepressant arm showed significantly higher rate of early response at 54%, X^2^ = 5.51 (1), p = 0.02, and rate of response at 33%, X^2^ = 9.64 (1), p = 0.002, but not in their remission rate of 13%, X^2^ = 0.05 (1), p = 0.82. With regards to general functional impairments (WSAS), participants in the iCBT arm had a significant decrease from baseline to final assessment by an average of 1.69 points (SD = 7.43) (9%), t(448) = 4.82, p < 0.001, d = 0.23, while those in the antidepressant arm reported an average reduction of 3.59 points (SD = 8.29) (16%), t(69) = 3.62, p < 0.001, d = 0.43. These differences across arms were not significant, t(511) = 1.59, p = 0.11. Furthermore, significant reductions in most other clinical symptoms were observed, in both treatment arms (all p<0.05), with the exception of schizotypy (p = 0.1) and impulsivity (p= 0.34) in the antidepressant arm.

Clinical Change Trajectories. A regression with QIDS-SR as the dependent variable and assessment week (0-4, within-subject) and treatment group (between subject) as independent variables was conducted. There was a linear effect of assessment week, β = -1.32, p < 0.001, indicating that symptoms decreased with time. There was a group main effect where symptoms were overall lower in the iCBT group (M = 11.7, SD = 4.77) than that in the antidepressant group (M = 12.4, SD = 5.25), β = -1.77, p < 0.001. There was a significant interaction between group and assessment week, β = 0.53, p < 0.001. Tests of simple effects revealed significant differences across the study arms in depression at baseline only (iCBT M = 13.60, SD = 4.18; antidepressant M = 16.40, SD = 3.96), β = -2.76, p < 0.001. There were no group differences in depression scores at any other timepoint (all p >= 0.42). For depression, there were significant reductions in symptoms week-on-week for the iCBT group (all p < 0.001), except for the final interval of week 3 to week 4 (β = -0.05, p = 0.72). Similar trends can be observed for the antidepressant group, where symptoms reduced week-on-week from baseline to the third weekly check-in (p <= 0.002). However, there was a slight increase in depression score from week 3 to week 4 where the final assessment took place (β = -0.67, p = 0.04). Participants in the antidepressant arm had larger improvements in depression for the baseline to week 1 interval only compared to iCBT (β = 2.33, p < 0.001), while all other week-to-week changes did not differ across groups (all p > 0.08).

## Comparison of baseline QIDS-SR symptomatology between dropouts and completers.

Sensitivity analyses comparing baseline QIDS-SR total scores between baseline completers (i.e., dropped out subsequent to completing the baseline assessment) and completers (completed all study assessments at all time points) revealed no significant differences between the two cohorts (drop outs M = 14.00, SD = 4.79; completers M = 14.30, SD = 4.37), t(708) = 0.59, p = 0.56. Similarly, there were no significant differences in baseline WSAS total scores between drop outs (M = 20.70, SD = 7.59) and completers (M = 19.50, SD = 6.79), t(694) = -1.62, p = 0.11.

## Inattentive responders on response consistency indicators.

We examined inattentive responders’ (N = 63) consistency in self-report height across two timepoints in the study (baseline and final assessments). Results were similar to that of the total sample, where the two height reports were highly correlated across the time points, r(61) = 94, p < 0.001. We also examined the internal consistency of self-report symptom assessments of inattentive responders. Cronbach’s alpha were good for all scales at baseline (i.e., range 0.74-0.96) and at final (i.e., range 0.75-0.96) at the final assessment.

# Qualitative Data Analysis

A qualitative content analysis was conducted on four open-ended free-text questions included in the online feedback survey. Content analysis is a qualitative research method used to analyse text-based, qualitative data through subjective interpretation of their content by way of systematic coding, categorising, and identifying themes or patterns (1). This method was deemed appropriate for the purpose of this analysis as it allows researchers to quantify concepts in the data by counting the number of times these concepts appeared, thus providing descriptive statistics fit for the quantitative reporting of this data. Two researchers reviewed the responses to each four of the survey questions independently and identified categories and sub-categories capturing common themes and ideas. A codebook describing each category/sub-category with inclusion/exclusion criteria was devised independently by the two researchers before they converged to discuss and agree on the list of categories/sub-categories. In the case where multiple themes emerged within one response, each distinct theme was categorised on their own, meaning respondents may have duplicated responses with specific elements pertaining to separate categories. Using the codebook, one of these researchers subsequently coded the responses and another independent research assistant separately coded 25% of responses to each question. Inter-rater reliability was checked using percentage agreement on the subset of 25% of responses to each question (2). Inter-rater reliability for each question exceeded 80%.

# References

1. Hsieh HF, Shannon SE. Three approaches to qualitative content analysis. Qual Health Res. 2005;15(9):1277-88.

2. McHugh ML. Interrater reliability: the kappa statistic. Biochem Medica. 2012;22(3):276.
