# Supplementary material for: The Precision in Psychiatry (PIP) study: Testing an internet-based methodology for accelerating research in treatment prediction and personalisation
Source: BMC Psychiatry. 2023 Jan 11;23:25. doi: 10.1186/s12888-022-04462-5 (PMC9832676; doi:10.1186/s12888-022-04462-5)
Supplement: Supplementary file 2 — Additional file 2. [file 12888_2022_4462_MOESM2_ESM.docx]

PIP Variable Directory

Table of Contents

[Socio-Demographics 4](#_Toc96612499)

[Age 4](#_Toc96612500)

[Sex 4](#_Toc96612501)

[Country of Residence 4](#_Toc96612502)

[Marital Status 4](#_Toc96612503)

[Education Level 4](#_Toc96612504)

[Employment Status 4](#_Toc96612505)

[Subjective Social Status 4](#_Toc96612506)

[Physical Health and Lifestyle 5](#_Toc96612507)

[Exercise 5](#_Toc96612508)

[Average days of exercise per week 5](#_Toc96612509)

[Average minutes of exercise on days of exercising 5](#_Toc96612510)

[Diet 5](#_Toc96612511)

[Overall diet quality 5](#_Toc96612512)

[Frequency of eating fish 5](#_Toc96612513)

[Frequency of taking diet supplements containing fish 5](#_Toc96612514)

[Drug Use 6](#_Toc96612515)

[Marijuana use 6](#_Toc96612516)

[Ecstasy/MDMA use 7](#_Toc96612517)

[Stimulant drug (e.g., cocaine, amphetamines) 8](#_Toc96612518)

[Opiates (e.g., on prescription; codeine, oxycodone) 9](#_Toc96612519)

[‘Street’ opiates (e.g., without prescription; heroin/codeine/oxycodone) 10](#_Toc96612520)

[Sedatives / Tranquilizers (e.g., on prescription) 11](#_Toc96612521)

[‘Street’ sedatives / Tranquilizers (e.g., without prescription) 12](#_Toc96612522)

[Physical Health Comorbidities (CIRS) 13](#_Toc96612523)

[Pain (PHQ-15) 13](#_Toc96612524)

[Smoking 13](#_Toc96612525)

[Cigarettes 13](#_Toc96612526)

[Vaping 14](#_Toc96612527)

[Other forms of tobacco 15](#_Toc96612528)

[Height and Weight 16](#_Toc96612529)

[Weight 16](#_Toc96612530)

[Height 16](#_Toc96612531)

[Psychosocial 16](#_Toc96612532)

[Stressful Life Events (SRRS) 16](#_Toc96612533)

[Childhood Trauma (CTQ) 16](#_Toc96612534)

[Perceived Social Support (MSPSS) 16](#_Toc96612535)

[Perceived Stress (PSS) 17](#_Toc96612536)

[Clinical 17](#_Toc96612537)

[Chronicity 17](#_Toc96612538)

[No. of poor mental health episodes 17](#_Toc96612539)

[Age of onset of mental health episodes 17](#_Toc96612540)

[Onset of current mental health episode 17](#_Toc96612541)

[Psychiatric Diagnoses (Self) 18](#_Toc96612542)

[Psychiatric Diagnosis (Family) 18](#_Toc96612543)

[Miscellaneous Psychiatric Symptoms 18](#_Toc96612544)

[Transdiagnostic Self-Report Questionnaires 18](#_Toc96612545)

[Apathy (AES) 18](#_Toc96612546)

[Alcoholism (AUDIT) 19](#_Toc96612547)

[Impulsivity (BIS) 19](#_Toc96612548)

[Eating disorder (EAT-26) 19](#_Toc96612549)

[Social anxiety (LSAS) 19](#_Toc96612550)

[Schizotypy (SSMS) 19](#_Toc96612551)

[Depression (SDS) 20](#_Toc96612552)

[State anxiety (STAI-T) 20](#_Toc96612553)

[Obsessive-Compulsive disorder (OCI-R) 20](#_Toc96612554)

[Clinical Outcome Measures 20](#_Toc96612555)

[Depression (QIDS-SR) 20](#_Toc96612556)

[Functional Impairment (WSAS) 21](#_Toc96612557)

[Treatment 21](#_Toc96612558)

[Treatment History 21](#_Toc96612559)

[Past antidepressant medication treatment 21](#_Toc96612560)

[No. of past antidepressant medication treatment* 21](#_Toc96612561)

[Success of past antidepressant medication treatment* 21](#_Toc96612562)

[Ever taken Sertraline* 21](#_Toc96612563)

[Blood test for antidepressant medication* 21](#_Toc96612564)

[Past psychotherapy treatment 22](#_Toc96612565)

[Types of past psychotherapy treatment* 22](#_Toc96612566)

[Success of past psychotherapy treatment* 22](#_Toc96612567)

[Failure of past psychotherapy treatment* 22](#_Toc96612568)

[Treatment Expectation 22](#_Toc96612569)

[Antidepressant Medication Type 23](#_Toc96612570)

[Selective Serotonin Reuptake Inhibitors (SSRIs) 23](#_Toc96612571)

[Serotonin and Norepinephrine Reuptake Inhibitors (SNRIs) 23](#_Toc96612572)

[Tricyclics 23](#_Toc96612573)

[Atypical Antidepressants 23](#_Toc96612574)

[Antidepressant Medication Dosage 23](#_Toc96612575)

[Antidepressant Medication Side Effects 24](#_Toc96612576)

[Presence of antidepressant side effects 24](#_Toc96612577)

[Types of antidepressant side effects 24](#_Toc96612578)

[Treatment Adherence 24](#_Toc96612579)

[Treatment adherence (antidepressant) 24](#_Toc96612580)

[Treatment adherence for (iCBT) 24](#_Toc96612581)

[Treatment non-adherence reasons (antidepressant) 24](#_Toc96612582)

[Treatment non-adherence reasons (iCBT) 25](#_Toc96612583)

[Extra Treatment Information 25](#_Toc96612584)

[Concurrent Medication Treatment 25](#_Toc96612585)

[Concurrent medication treatment type (antidepressant)* 25](#_Toc96612586)

[Concurrent medication treatment dosage (antidepressant)* 26](#_Toc96612587)

[Concurrent medication treatment type (iCBT)* 26](#_Toc96612588)

[Concurrent Psychotherapy Treatment 26](#_Toc96612589)

[Concurrent psychotherapy treatment (baseline) 26](#_Toc96612590)

[Types of concurrent psychotherapy treatment (baseline)* 26](#_Toc96612591)

[Concurrent psychotherapy treatment (weekly) 27](#_Toc96612592)

[Concurrent psychotherapy treatment type (weekly)* 27](#_Toc96612593)

[iCBT Treatment Engagement 27](#_Toc96612594)

[Total time spent (seconds) 27](#_Toc96612595)

[Number of sessions 27](#_Toc96612596)

[Average session length (seconds) 28](#_Toc96612597)

[Number of activities 28](#_Toc96612598)

[Number of modules in program 28](#_Toc96612599)

[Modules completed 28](#_Toc96612600)

[Percentage of program viewed 28](#_Toc96612601)

[Activities per session 28](#_Toc96612602)

[Modules per session 28](#_Toc96612603)

[Number of journal entries 28](#_Toc96612604)

[Length of journal entries 29](#_Toc96612605)

[Supporter Experience 29](#_Toc96612606)

[Number of reviews 29](#_Toc96612607)

[Length of reviews 29](#_Toc96612608)

[Average review length 29](#_Toc96612609)

[Reviews fit versus template 29](#_Toc96612610)

[Number of review notes 29](#_Toc96612611)

[Review note length 30](#_Toc96612612)

[Baseline Depression Score 30](#_Toc96612613)

[Baseline Anxiety Score 30](#_Toc96612614)

[Number of tools used 30](#_Toc96612615)

[Modules viewed 30](#_Toc96612616)

[Mode of Engagement 30](#_Toc96612617)

[Site 31](#_Toc96612618)

[SilverCloud Program Name 31](#_Toc96612619)

[Cognitive Performance 31](#_Toc96612620)

[Perceptual Decision-Making Task 31](#_Toc96612621)

[Two-Step Reinforcement-Learning Task 32](#_Toc96612622)

[Learning Under Volatility Task 33](#_Toc96612623)

[Abstract Reasoning Test 34](#_Toc96612624)

[Data Quality Probes 34](#_Toc96612625)

[Distraction Probe 34](#_Toc96612626)

[Substance Use Probe 35](#_Toc96612627)

[Attention Check OCI 35](#_Toc96612628)

[Attention Check WSAS 35](#_Toc96612629)

[References 35](#_Toc96612630)

# Socio-Demographics

##

## Age

Variable type: Continuous discrete variable

Measurement: “What is your age?”

Response options: 18-70 (years of age)

Variable name: age

## Sex

Variable type: Nominal variable

Measurement: “What is your sex?”

Response options: Female (0), Male (1), Intersex (2), Male to Female (3), Female to Male (4)

Variable name: Sex

## Country of Residence

Variable type: Nominal variable

Measurement: “What is your country of residence?”

Response options: Free-entry field

Variable name: Country

## Marital Status

Variable type: Ordinal variable

Measurement: “What is your marital status?”

Response options: Single (0), Married (1), In a relationship (2), Separated (3), Divorced (4), Widowed (5)

Variable name: MStatus

## Education Level

Variable type: Ordinal variable

Measurement: “What is the highest level of education that you’ve completed?”

Response options: No schooling completed (0), Some early primary (1), Completed primary school (2), some secondary education (3), completed secondary education (4), trade/technical/vocational training (5), some undergraduate education (6), completed undergraduate education (7), some postgraduate education (8), master's degree (9), doctorate degree (10)

Variable name: Education

## Employment Status

Variable type: Ordinal variable

Measurement: “What best describes your current employment status?”

Response options: Unemployed not looking (0), unemployed looking (1), full-time employed (2), part-time employed (3), self-employed (4), retired (5)

Variable name: Employment

## Subjective Social Status

Variable type: Continuous discrete variable

Measurement: MacArthur Scale of Subjective Social Status (1)

Response options: 0-10 (the higher the number, the higher the subjective status)

Variable name: SSStatus

# Physical Health and Lifestyle

##

## Exercise

### Average days of exercise per week

Variable type: Continuous discrete variable

Measurement: “On average, how many days per week do you engage in moderate to strenuous exercise? (e.g. a brisk walk, a run, swimming, cycling, weight lifting, team sports etc.)”. Item drawn from the Physical Activity Vital Sign (PAVS)

Response options: 0-7 days

Variable name: Exercise_days

### Average minutes of exercise on days of exercising

Variable type: Continuous discrete variable

Measurement: “On these days, on average, how many minutes do you engage in exercise at this level?”

Response options: 0 min, 10 min, 20 min, 30 min, 40 min, 50 min, 60+ min

Variable name: Exercise_min

## Diet

### Overall diet quality

Variable type: Ordinal variable

Measurement: “In general, how healthy is your overall diet?”

Response options: Very poor (0), Poor (1), Fair (2), Good (3), Very good (4), Excellent (5)

Variable name: Diet_quality

### Frequency of eating fish

Variable type: Ordinal variable

Measurement: “How often do you usually eat fresh or canned fish? (NOT including fish and chips)”

Response options: rarely/never (0), once a month (1), twice a month (2), once a week (3), twice a week (4), every second day (5), once a day (6), more than once a day (7)

Variable name: Diet_fish

### Frequency of taking diet supplements containing fish

Variable type: Ordinal variable

Measurement: “Do you regularly take diet supplements that contain fish oils or omega 3 fatty acids?”

Response options: rarely/never (0), once a month (1), twice a month (2), once a week (3), twice a week (4), every second day (5), once a day (6), more than once a day (7)

Variable name: Diet_fishsupp

## Drug Use

### Marijuana use

Variable type: Ordinal variable

Measurement: “Have you ever taken marijuana?”

Response options: No (0), Yes (1)

Variable name: Drugs_1

#### Marijuana use habits

*Conditional on (1) in Drugs_1

Variable type: Ordinal variable

Measurement: “Have you ever taken marijuana?”

Response options: Once/rarely in the past (0), Frequently in the past but quit (1), Still ongoing (2)

Variable name: Drugs_1_group

#### Frequency of marijuana use in the past (quitters)

*Conditional on (1) in Drugs_1_group

Variable type: Ordinal variable

Measurement: “How frequently did you take it in the past?”

Response options: Less than once a month (0), Every month (1), Every week (2), Daily or almost daily (3), More than once a day (4)

Variable name: Drugs_1_past_freq

#### Age when first started using marijuana (quitters)

*Conditional on (1) in Drugs_1_group

Variable type: Continuous discrete variable

Measurement: “How old were you when you started taking it?”

Response options: 0-70 years old

Variable name: Drugs_1_past_age1

#### Age when quitted using marijuana (quitters)

*Conditional on (1) in Drugs_1_group

Variable type: Continuous discrete variable

Measurement: “How old were you when you quit?”

Response options: 0-70 years old

Variable name: Drugs_1_past_age2

#### Frequency of current marijuana use (users)

*Conditional on (2) in Drugs_1_group

Variable type: Ordinal variable

Measurement: “How frequently do you take it?”

Response options: Less than once a month (0), Every month (1), Every week (2), Daily or almost daily (3), More than once a day (4)

Variable name: Drugs_1_freq

#### Age when first started using marijuana (users)

*Conditional on (2) in Drugs_1_group

Variable type: Continuous discrete variable

Measurement: “How old were you when you started taking it?”

Response options: 0-70 years old

Variable name: Drugs_1_age

### Ecstasy/MDMA use

Variable type: Ordinal variable

Measurement: “Have you ever taken ecstasy/MDMA?”

Response options: No (0), Yes (1)

Variable name: Drugs_2

#### Ecstasy/MDMA use habits

*Conditional on (1) in Drugs_2

Variable type: Ordinal variable

Measurement: “Have you ever taken ecstasy/MDMA?”

Response options: Once/rarely in the past (0), Frequently in the past but quit (1), Still ongoing (2)

Variable name: Drugs_2_group

#### Frequency of ecstasy/MDMA use in the past (quitters)

*Conditional on (1) in Drugs_2_group

Variable type: Ordinal variable

Measurement: “How frequently did you take it in the past?”

Response options: Less than once a month (0), Every month (1), Every week (2), Daily or almost daily (3), More than once a day (4)

Variable name: Drugs_2_past_freq

#### Age when first started using ecstasy/MDMA (quitters)

*Conditional on (1) in Drugs_2_group

Variable type: Continuous discrete variable

Measurement: “How old were you when you started taking it?”

Response options: 0-70 years old

Variable name: Drugs_2_past_age1

#### Age when quitted using ecstasy/MDMA (quitters)

*Conditional on (1) in Drugs_2_group

Variable type: Continuous discrete variable

Measurement: “How old were you when you quit?”

Response options: 0-70 years old

Variable name: Drugs_2_past_age2

#### Frequency of current ecstasy/MDMA use (users)

*Conditional on (2) in Drugs_2_group

Variable type: Ordinal variable

Measurement: “How frequently do you take it?”

Response options: Less than once a month (0), Every month (1), Every week (2), Daily or almost daily (3), More than once a day (4)

Variable name: Drugs_2_freq

#### Age when first started using ecstasy/MDMA (users)

*Conditional on (2) in Drugs_2_group

Variable type: Continuous discrete variable

Measurement: “How old were you when you started taking it?”

Response options: 0-70 years old

Variable name: Drugs_2_age

### Stimulant drug (e.g., cocaine, amphetamines)

Variable type: Ordinal variable

Measurement: “Have you ever taken stimulant drugs (e.g., cocaine/amphetamines)?”

Response options: No (0), Yes (1)

Variable name: Drugs_3

#### Stimulant drug use habits

*Conditional on (1) in Drugs_3

Variable type: Ordinal variable

Measurement: “Have you ever taken stimulant drugs (e.g., cocaine/amphetamines)?”

Response options: Once/rarely in the past (0), Frequently in the past but quit (1), Still ongoing (2)

Variable name: Drugs_3_group

#### Frequency of stimulant drug use in the past (quitters

*Conditional on (1) in Drugs_3_group

Variable type: Ordinal variable

Measurement: “How frequently did you take it in the past?”

Response options: Less than once a month (0), Every month (1), Every week (2), Daily or almost daily (3), More than once a day (4)

Variable name: Drugs_3_past_freq

#### Age when first started using stimulant drug (quitters)

*Conditional on (1) in Drugs_3_group

Variable type: Continuous discrete variable

Measurement: “How old were you when you started taking it?”

Response options: 0-70 years old

Variable name: Drugs_3_past_age1

#### Age when quitted using stimulant drug (quitters)

*Conditional on (1) in Drugs_3_group

Variable type: Continuous discrete variable

Measurement: “How old were you when you quit?”

Response options: 0-70 years old

Variable name: Drugs_3_past_age2

#### Frequency of current stimulant drug use (users)

*Conditional on (2) in Drugs_3_group

Variable type: Ordinal variable

Measurement: “How frequently do you take it?”

Response options: Less than once a month (0), Every month (1), Every week (2), Daily or almost daily (3), More than once a day (4)

Variable name: Drugs_3_freq

#### Age when first started using stimulant drug (users)

*Conditional on (2) in Drugs_3_group

Variable type: Continuous discrete variable

Measurement: “How old were you when you started taking it?”

Response options: 0-70 years old

Variable name: Drugs_3_age

### Opiates (e.g., on prescription; codeine, oxycodone)

Variable type: Ordinal variable

Measurement: “Have you ever taken opiates on prescription excluding Solpadeine and Neurofen Plus (e.g. codeine, oxycodone)?”

Response options: No (0), Yes (1)

Variable name: Drugs_4

#### Opiate use habits

*Conditional on (1) in Drugs_4

Variable type: Ordinal variable

Measurement: “Have you ever taken opiates on prescription excluding Solpadeine and Neurofen Plus (e.g. codeine, oxycodone)?”

Response options: Once/rarely in the past (0), Frequently in the past but quit (1), Still ongoing (2)

Variable name: Drugs_4_group

#### Frequency of opiate use in the past (quitters)

*Conditional on (1) in Drugs_4_group

Variable type: Ordinal variable

Measurement: “How frequently did you take it in the past?”

Response options: Less than once a month (0), Every month (1), Every week (2), Daily or almost daily (3), More than once a day (4)

Variable name: Drugs_4_past_freq

#### Age when first started using opiates (quitters)

*Conditional on (1) in Drugs_4_group

Variable type: Continuous discrete variable

Measurement: “How old were you when you started taking it?”

Response options: 0-70 years old

Variable name: Drugs_4_past_age1

#### Age when quitted using opiates (quitters)

*Conditional on (1) in Drugs_4_group

Variable type: Continuous discrete variable

Measurement: “How old were you when you quit?”

Response options: 0-70 years old

Variable name: Drugs_4_past_age2

#### Frequency of current opiate use (users)

*Conditional on (2) in Drugs_4_group

Variable type: Ordinal variable

Measurement: “How frequently do you take it?”

Response options: Less than once a month (0), Every month (1), Every week (2), Daily or almost daily (3), More than once a day (4)

Variable name: Drugs_4_freq

#### Age when first started using opiates (users)

*Conditional on (2) in Drugs_4_group

Variable type: Continuous discrete variable

Measurement: “How old were you when you started taking it?”

Response options: 0-70 years old

Variable name: Drugs_4_age

### ‘Street’ opiates (e.g., without prescription; heroin/codeine/oxycodone)

Variable type: Ordinal variable

Measurement: “Have you ever taken ‘street’ opiates, that is, without prescription (e.g. heroine/codeine/oxycodone)?”

Response options: No (0), Yes (1)

Variable name: Drugs_5

#### Street opiate use habits

*Conditional on (1) in Drugs_5

Variable type: Ordinal variable

Measurement: “Have you ever taken ‘street’ opiates, that is, without prescription (e.g. heroine/codeine/oxycodone)?”

Response options: Once/rarely in the past (0), Frequently in the past but quit (1), Still ongoing (2)

Variable name: Drugs_5_group

#### Frequency of street opiate use in the past (quitters)

*Conditional on (1) in Drugs_5_group

Variable type: Ordinal variable

Measurement: “How frequently did you take it in the past?”

Response options: Less than once a month (0), Every month (1), Every week (2), Daily or almost daily (3), More than once a day (4)

Variable name: Drugs_5_past_freq

#### Age when first started using street opiates (quitters)

*Conditional on (1) in Drugs_5_group

Variable type: Continuous discrete variable

Measurement: “How old were you when you started taking it?”

Response options: 0-70 years old

Variable name: Drugs_5_past_age1

#### Age when quitted using street opiates (quitters)

*Conditional on (1) in Drugs_5_group

Variable type: Continuous discrete variable

Measurement: “How old were you when you quit?”

Response options: 0-70 years old

Variable name: Drugs_5_past_age2

#### Frequency of current street opiate use (users)

*Conditional on (2) in Drugs_5_group

Variable type: Ordinal variable

Measurement: “How frequently do you take it?”

Response options: Less than once a month (0), Every month (1), Every week (2), Daily or almost daily (3), More than once a day (4)

Variable name: Drugs_5_freq

#### Age when first started using street opiates (users)

*Conditional on (2) in Drugs_5_group

Variable type: Continuous discrete variable

Measurement: “How old were you when you started taking it?”

Response options: 0-70 years old

Variable name: Drugs_5_age

### Sedatives / Tranquilizers (e.g., on prescription)

Variable type: Ordinal variable

Measurement: “Have you ever taken sedatives/tranquilizers on prescription?”

Response options: No (0), Yes (1)

Variable name: Drugs_6

#### Sedative / tranquilizer use habits

*Conditional on (1) in Drugs_6

Variable type: Ordinal variable

Measurement: “Have you ever taken sedatives/tranquilizers on prescription?”

Response options: Once/rarely in the past (0), Frequently in the past but quit (1), Still ongoing (2)

Variable name: Drugs_6_group

#### Frequency of sedative / tranquilizer use in the past (quitters)

*Conditional on (1) in Drugs_6_group

Variable type: Ordinal variable

Measurement: “How frequently did you take it in the past?”

Response options: Less than once a month (0), Every month (1), Every week (2), Daily or almost daily (3), More than once a day (4)

Variable name: Drugs_6_past_freq

#### Age when first started using sedative / tranquilizer (quitters)

*Conditional on (1) in Drugs_6_group

Variable type: Continuous discrete variable

Measurement: “How old were you when you started taking it?”

Response options: 0-70 years old

Variable name: Drugs_6_past_age1

#### Age when quitted using sedative / tranquilizer (quitters)

*Conditional on (1) in Drugs_6_group

Variable type: Continuous discrete variable

Measurement: “How old were you when you quit?”

Response options: 0-70 years old

Variable name: Drugs_6_past_age2

#### Frequency of current sedative / tranquilizer use (users)

*Conditional on (2) in Drugs_6_group

Variable type: Ordinal variable

Measurement: “How frequently do you take it?”

Response options: Less than once a month (0), Every month (1), Every week (2), Daily or almost daily (3), More than once a day (4)

Variable name: Drugs_6_freq

#### Age when first started using sedative / tranquilizer (users)

*Conditional on (2) in Drugs_6_group

Variable type: Continuous discrete variable

Measurement: “How old were you when you started taking it?”

Response options: 0-70 years old

Variable name: Drugs_6_age

### ‘Street’ sedatives / Tranquilizers (e.g., without prescription)

Variable type: Ordinal variable

Measurement: “Have you ever taken street’ sedatives / tranquilizers, that is, without prescription?”

Response options: No (0), Yes (1)

Variable name: Drugs_7

#### Street sedative / tranquilizer use habits

*Conditional on (1) in Drugs_7

Variable type: Ordinal variable

Measurement: “Have you ever taken ‘street’ sedatives / tranquilizers, that is, without prescription?”

Response options: Once/rarely in the past (0), Frequently in the past but quit (1), Still ongoing (2)

Variable name: Drugs_7_group

#### Frequency of street sedative / tranquilizer use in the past (quitters)

*Conditional on (1) in Drugs_7_group

Variable type: Ordinal variable

Measurement: “How frequently did you take it in the past?”

Response options: Less than once a month (0), Every month (1), Every week (2), Daily or almost daily (3), More than once a day (4)

Variable name: Drugs_7_past_freq

#### Age when first started using street sedative / tranquilizer (quitters)

*Conditional on (1) in Drugs_7_group

Variable type: Continuous discrete variable

Measurement: “How old were you when you started taking it?”

Response options: 0-70 years old

Variable name: Drugs_7_past_age1

#### Age when quitted using street sedative / tranquilizer (quitters)

*Conditional on (1) in Drugs_7_group

Variable type: Continuous discrete variable

Measurement: “How old were you when you quit?”

Response options: 0-70 years old

Variable name: Drugs_7_past_age2

#### Frequency of current street sedative / tranquilizer use (users)

*Conditional on (2) in Drugs_7_group

Variable type: Ordinal variable

Measurement: “How frequently do you take it?”

Response options: Less than once a month (0), Every month (1), Every week (2), Daily or almost daily (3), More than once a day (4)

Variable name: Drugs_7_freq

#### Age when first started using street sedative / tranquilizer (users)

*Conditional on (2) in Drugs_7_group

Variable type: Continuous discrete variable

Measurement: “How old were you when you started taking it?”

Response options: 0-70 years old

Variable name: Drugs_7_age

## Physical Health Comorbidities (CIRS)

Variable type: Continuous discrete variable

Measurement: Cumulative Illness Rating Scale (CIRS) (2)

Response options: A 6-item Likert scale for 13 items: None (0), Mild (1), Moderate (2), Severe (3), Extremely severe (4). A total score is yielded by summing item responses. The higher the score, the higher the impairment.

Variable name: CIRS_total

## Pain (PHQ-15)

Variable type: Continuous discrete variable

Measurement: Patient Health Questionnaire (PHQ-15) (3)

Response options: A 3-item Likert Scale for 5 items. Not bothered at all (0), Bothered a little (1), Bothered a lot (2). A total score is yielded by summing item responses. The higher the score, the higher the pain level.

Variable name: PAIN_total

## Smoking

### Cigarettes

#### Ever smoked cigarettes

Variable type: Binary variable

Measurement: “Have you ever smoked cigarettes?”

Response options: No (0), Yes (1)

Variable name: Smoking_ever

#### Smoking habits (users or quitters)

*Conditional on (1) in Smoking_ever

Variable type: Ordinal variable

Measurement: “Please select the option which best applies to you”

Response options: Once or rare occasions in the past (0), Frequently in the past (1), Ongoing basis (2)

Variable name: Smoking_group

#### Frequency of smoking in the past (quitters)

*Conditional on (1) in Smoking_group

Variable type: Ordinal variable

Measurement: “How frequently did you smoke in the past? (include instances where you have only taken one or two puffs”

Response options: Less than once per day (0), 1-5 times (1), 5-10 times (2), 10-20 times (3), 20-30 times (4), more than 30 times (5)

Variable name: Smoking_past_freq

#### Age when first started smoking (quitters)

*Conditional on (1) in Smoking_group

Variable type: Continuous discrete variable

Measurement: “How old were you when you started smoking?”

Response options: 0-70 years old

Variable name: Smoking_past_AgeStart

#### Age when quitted smoking (quitters)

*Conditional on (1) in Smoking_group

Variable type: Continuous discrete variable

Measurement: “How old were you when you quit?”

Response options: 0-70 years old

Variable name: Smoking_past_AgeQuit

#### Frequency of current smoking (users)

*Conditional on (2) in Smoking_group

Variable type: Ordinal variable

Measurement: “How frequently do you smoke? (include instances where you have only taken one or two puffs)

Response options: Less than once per day (0), 1-5 times (1), 5-10 times (2), 10-20 times (3), 20-30 times (4), more than 30 times (5)

Variable name: Smoking_present_freq

#### Age when first started smoking (users)

*Conditional on (2) in Smoking_group

Variable type: Continuous discrete variable

Measurement: “How old were you when you started smoking?”

Response options: 0-70 years old

Variable name: Smoking_present_AgeStart

### Vaping

#### Ever vaped (i.e., smoked e-cigarettes)

Variable type: Binary variable

Measurement: “Have you ever vaped (i.e., smoked e-cigarettes)?”

Response options: No (0), Yes (1)

Variable name: Smoking_vape_ever

#### Vaping habits

*Conditional on (1) on Smoking_vape_ever

Variable type: Ordinal variable

Measurement: “Please select the option which best applies to you:”

Response options: Once or rare occasions in the past (0), Frequently in the past (1), Ongoing basis (2)

Variable name: Smoking_vape_group

#### Frequency of vaping in the past (quitters)

*Conditional on (1) on Smoking_vape_group

Variable type: Ordinal variable

Measurement: “How frequently did you vape in the past? (account for all instances – even times where you have only taken one or two puffs)

Response options: Less than once per day (0), 1-5 times (1), 5-10 times (2), 10-20 times (3), 20-30 times (4), more than 30 times (5)

Variable name: Smoking_vape_past_freq

#### Age when first started vaping (quitters)

*Conditional on (1) on Smoking_vape_group

Variable type: Continuous discrete variable

Measurement: “How old were you when you started vaping?”

Response options: 0-70 years old

Variable name: Smoking_vape_past_AgeStart

#### Age when quitted vaping (quitters)

*Conditional on (1) on Smoking_vape_group

Variable type: Continuous discrete variable

Measurement: “How old were you when you quit?”

Response options: 0-70 years old

Variable name: Smoking_vape_past_AgeQuit

#### Frequency of current vaping (users)

*Conditional on (2) on Smoking_vape_group

Variable type: Ordinal variable

Measurement: “How frequently do you vape? (account for all instances – even times when you have only taken one or two puffs)

Response options: Less than once per day (0), 1-5 times (1), 5-10 times (2), 10-20 times (3), 20-30 times (4), more than 30 times (5)

Variable name: Smoking_vape_present_freq

#### Age when first started vaping (users)

*Conditional on (2) on Smoking_vape_group

Variable type: Continuous discrete variable

Measurement: “How old were you when you started vaping?”

Response options: 0-70 years old

Variable name: Smoking_vape_present_AgeStart

### Other forms of tobacco

Variable type: Binary variable

Measurement: “Do you consume other forms of tobacco (e.g. chewing tobacco, snuff) on a regular basis?”

Response options: Yes (1), No (0)

Variable name: Smoking_Other

## Height and Weight

### Weight

Variable type: Continuous discrete variable

Measurement: “What is your weight at present (in lbs) (please give your best estimate)”

Response options: Free numeric entry field

Variable name: currentweight

### Height

#### Height in feet

Variable type: Continuous discrete variable

Measurement: “What is your height (in feet and inches)?”

Response options: 1-8 feet

Variable name: FeetDropdownld

#### Height in inches

Variable type: Continuous discrete variable

Measurement: “What is your height (in feet and inches)?”

Response options: 0-11 inches

Variable name: InchesDropdownld

# Psychosocial

## Stressful Life Events (SRRS)

Variable type: Continuous discrete variable

Measurement: Social Readjustment Rating Scale (SRRS) (4)

Response options: False (0), True (1) for 43 items. A total score is yielded by multiplying the ‘True’ answers by a specific weight. Some life events are thus classes as more stressful than others (e.g., death of a spouse adds 100 points to total, whereas a change in residence adds 20). The higher the summed score, the more stressful events.

Variable name: SRRS_total

## Childhood Trauma (CTQ)

Variable type: Continuous discrete variables

Measurement: Childhood Trauma Questionnaire (CTQ) (5)

Response options: A 5-item Likert scale for 28 items: Never true (0), Rarely true (1), Sometimes true (2), Often true (3), Very often true (4). A total score and 5 subscale scores are yielded by summing respective item responses. The higher the score, the higher the childhood trauma.

Variable name: CTQ_total, CTQ_emabuse, CTQ_physabuse, CTQ_sexabuse, CTQ_emneglect, CTQ_physneglect

## Perceived Social Support (MSPSS)

Variable type: Continuous discrete variable

Measurement: Multidimensional Scale of Perceived Social Support (MSPSS) (6)

Response options: A 7-item Likert scale for 12 items: Very strongly disagree (0), Strongly disagree (1), Mildly disagree (2), Neutral (3), Mildly agree (4), Strongly Agree (5), Very strongly Agree (6). A total score is yielded by summing item responses. The higher the score, the higher the perceived support.

Variable name: SOSU_total

## Perceived Stress (PSS)

Variable type: Continuous discrete variable

Measurement: Perceived Stress Scale (PSS) (7)

Response options: A 5-item Likert scale for 10 items: Never (0), Almost Never (1), Sometimes (2), Fairly often (3), Very often (4). A total score is yielded by summing item responses. The higher the score, the more perceived stress.

Variable name: PSS_total

# Clinical

## Chronicity

### No. of poor mental health episodes^[[1]](#footnote-2)^

Variable type: Continuous discrete variable

Measurement: “How many times in your life have you experienced an episode of poor mental health?”

Response options: 0-29 times

Variable name: TFLTE_episodes

### Age of onset of mental health episodes

Variable type: Continuous discrete variable

Measurement: “What age were you when you experienced your first episode of poor mental health?”

Response options: 0-70 years

Variable name: TFLTE_ageonset

### Onset of current mental health episode^[[2]](#footnote-3)^

Variable type: Continuous discrete variable

Measurement: “When did this current episode of poor mental health start?”

Response options: DD/MM/YY

Variable name: TFLTE_currentonset

## Psychiatric Diagnoses (Self)

Variable type: Binary variables

Measurement: “I currently have a diagnosis of… (Please select all that apply)”

Response options: Yes (TRUE) and No (FALSE) for 13 diagnoses. A total score is yielded by summing the number of ‘True’ for all diagnoses.

Variable name: Diagnoses_total, Diagnosis_Dep, Diagnosis_OCD, Diagnosis_GAD, Diagnosis_PanD, Diagnosis_PTSD, Diagnosis_BPD, Diagnosis_Schiz, Diagnosis_PersD, Diagnosis_SubD, Diagnosis_AnoD, Diagnosis_BulD, Diagnosis_BinD, Diagnosis_TicD

## Psychiatric Diagnosis (Family)

Variable type: Continuous discrete variable

Measurement: “Do you have any close relatives who ever received a diagnosis of a mental health disorder? (Please only count your biological parents, biological siblings or biological children)”

Response options: 0-10 close relatives

Variable name: TFLTE_famHist

## Miscellaneous Psychiatric Symptoms

Variable type: Continuous discrete variable

Measurement: Top predictors in Chekroud and colleagues’ 2016 study (8)

Response options: Yes (1,) No (0) for 8 items.

- Have you experienced depressed mood most of the day, nearly every day?
- Have you been bothered by aches and pains in many different parts of your body?
- Did reminders of a traumatic event make you shake, break out into a sweat, or have a racing heart?
- Did you try to avoid activities, places, or people that reminded you of a traumatic event?
- Did you have attacks of anxiety that caused you to avoid certain situations or to change your behaviour or normal routine?
- Did standing in long lines make you feel fearful, anxious, or nervous?
- Did driving or riding in a car make you feel fearful, anxious, or nervous?
- Have you ever witnessed a traumatic event such as rape, assault, someone dying in an accident, or any other extremely upsetting incident?

## Transdiagnostic Self-Report Questionnaires

### Apathy (AES)^[[3]](#footnote-4)^

Variable type: Continuous discrete variable

Measurement: Apathy Evaluation Scale (AES) (9)

Response options: A 4-point Likert scale for 18 items: Not at all (0), Slightly (1), Somewhat (2), A lot (3). A total score is yielded by summing item responses. The higher the score, the higher apathetic.

Variable name: AES_total

### Alcoholism (AUDIT)

Variable type: Continuous discrete variable

Measurement: Alcohol Use Disorder Identification Test (AUDIT; Saunders et al., 1993)

Response options: A 5-point Likert Scale for 8 items (0-4) and a 3-point Likert Scale for 2 items. A total score is yielded by summing item responses. The higher the score, the higher the likelihood of hazardous/harmful alcohol consumption.

Variable name: AUDIT_total

### Impulsivity (BIS)

Variable type: Continuous discrete variable

Measurement: Barratt Impulsivity Scale (BIS) (10)

Response options: A 4-point Likert scale for 30 items: Rarely/Never (1), Occasionally (2), Often 3), Almost always/Always (4). A total score is yielded by summing item responses. The higher the score, the higher the level of impulsivity.

Variable name: BIS_total

### Eating disorder (EAT-26)

Variable type: Continuous discrete variable

Measurement: Eating Attitudes Test (EAT-26) (11)

Response options: A 6-point Likert scale for 26 items: Never (1), Rarely (2), Sometimes (3), Often (4), Usually (5), Always (6). A total score is yielded by summing item responses. The higher the score, the higher the level of higher level of concern about dieting, body weight, or problematic eating behaviours.

Variable name: EAT_total

### Social anxiety (LSAS)^[[4]](#footnote-5)^

Variable type: Continuous discrete variable

Measurement: Liebowitz Social Anxiety Scale (LSAS) (12)

Response options: A 4-point Likert scale for 24 items: None (0), Mild (1), Moderate (2), Severe (3). A total score is yielded by summing item responses. The higher the score, the higher the level of social anxiety.

Variable name: LSAS_total

### Schizotypy (SSMS)

Variable type: Continuous discrete variable

Measurement: Short Scales for Measuring Schizotypy (SSMS-R) (13)

Response options: No (0) and Yes (1) for 43 items. A total score is yielded by summing

‘item responses. The higher the score, the likely the presence of schizotypy.

Variable name: SCZ_total

### Depression (SDS)

Variable type: Continuous discrete variable

Measurement: Self-Rating Depression Scale (SDS) (14)

Response options: A 4 Likert scale for 20 items: A little of the time (1), Some of the time (2), Good part of the time (3), Most of the time (4). A total score is yielded by summing item responses. The higher the score, the more depressed the individual.

Variable name: SDS_total

### State anxiety (STAI-T)^[[5]](#footnote-6)^

Variable type: Continuous discrete variable

Measurement: State-Trait Anxiety Inventory (STAI) (15)

Response options: A 4-point Likert scale for 20 items: Not at all (1), Somewhat (2), Moderately so (3), Very much so (4). A total score is yielded by summing item responses. The higher the score, the higher the level of anxiety.

Variable name: STAI_total

### Obsessive-Compulsive disorder (OCI-R)

Variable type: Continuous discrete variable

Measurement: Revised Obsessive-Compulsive Inventory (OCI-R) (16)

Response options: A 5-point Likert scale for 18 items: Not at all (0), A little (1), Moderately (2), A lot (3), Extremely (4). A total score is yielded by summing item responses. The higher the score, the more likely the presence of OCD.

Variable name: OCI_total

## Clinical Outcome Measures

### Depression (QIDS-SR)^[[6]](#footnote-7)^

Variable type: Continuous discrete variable

Measurement: Quick Inventory of Depressive Symptomatology – Self-Report (QIDS-SR) (17)

Response options: A 4-point Likert scale for 16 items (0 to 3). A total score is yielded by summing 9 item responses. The higher the score, the higher the level of depression.

Variable name: QIDS_total

### Functional Impairment (WSAS)

Variable type: Continuous discrete variable

Measurement: Work and Social Adjustment Scale (WSAS; Mundt et al., 2002)

Response options: A 8-point Likert scale for 5 items: Not at all (0), Slightly (2), Definitely (4), Markedly (6), Very severely (8). A total score is yielded by summing item responses. The higher the score, the higher the level of impairment.

Variable name: WSAS_total

# Treatment

## Treatment History

### Past antidepressant medication treatment

Variable type: Binary variable

Measurement: “Have you ever in the past completed a course of antidepressant medication?”

Response options: No (0), Yes (1)

Variable name: TFLTE_ADpast

### No. of past antidepressant medication treatment*

*Conditional on TRUE in TFLTE_ADpast

Variable type: Continuous discrete variable

Measurement: “How many times?”

Response options: 0-10 times

Variable name: TFLTE_ADpast_freq

### Success of past antidepressant medication treatment*

*Conditional on TRUE in TFLTE_ADpast

Variable type: Continuous discrete variable

Measurement: “How many times would you say this kind of treatment has resulted in a significant improvement in your symptoms? “

Response options: 0-10 times

Variable name: TFLTE_ADpast_helped

### Ever taken Sertraline*

*Conditional on TRUE in TFLTE_ADpast

Variable type: Binary variable

Measurement: “Have you ever taken an antidepressant called Sertraline (also known as Zoloft/Lustral)?”

Response options: No (0), Yes (1)

Variable name: TFLTE_ADpast_sertraline

### Blood test for antidepressant medication*

*Conditional on TRUE in TFLTE_ADpast

Variable type: Binary variable

Measurement: “Did your prescriber take a blood sample from you before they gave you the prescription for your antidepressant?”

Response options: No (0), Yes (1)

Variable name: TFLTE_ADpast_bloodTest

### Past psychotherapy treatment

Variable type: Binary variable

Measurement: “Have you ever in the past completed a course of psychological therapy?”

Response options: No (0), Yes (1)

Variable name: TFLTE_PTpast

### Types of past psychotherapy treatment*

*Conditional on TRUE in TFLTE_PTpast

Variable type: Binary variables

Measurement: “Please select the type of psychological therapy:”

Response options: Yes (TRUE), No (FALSE) for 10 psychotherapy interventions

Variable name: 10 variables with TFLTE_PTpast the short-handed name of each psychotherapy

- Behavioural therapy (TFLTE_PTpast_BT)
- Cognitive Behavioural Therapy (TFLTE_PTpast_CBT)
- Counselling (TFLTE_PTpast_Counsig)
- Cognitive Therapy (TFLTE_PTpast_CT)
- Family Therapy (TFLTE_PTpast_FT)
- Interpersonal Therapy (TFLTE_PTpast_IPT)
- Mindfulness (TFLTE_PTpast_Mindflns)
- Other (TFLTE_PTpast_Other)
- Psychoanalytic (TFLTE_PTpast_Psychoan)
- Psychodynamic (TFLTE_PTpast_Psychodyn)

### Success of past psychotherapy treatment*

*Conditional on TRUE in TFLTE_PTpast

Variable type: Continuous discrete variable

Measurement: “How many times would you say this kind of treatment has resulted in a significant improvement in your symptoms?”

Response options: 0-10 times

Variable name: TFLTE_PTpast_helped

### Failure of past psychotherapy treatment*

*Conditional on TRUE in TFLTE_PTpast

Variable type: Continuous discrete variable

Measurement: “How many times would you say this kind of treatment has failed to result in a significant improvement in your symptoms?”

Response options: 0-10 times

Variable name: TFLTE_PTpast_failed

## Treatment Expectation

Variable type: Ordinal variable

Measurement: “What point on this 10-point scale best describes your expectations about what is likely to happen as a result of your current antidepressant treatment?”

Response options: 10-point Likert scale from “I don’t expect to feel any better" (0) to "I expect to feel completely better" (9)

Variable name: TFLTE_expectations

## Antidepressant Medication Type

Variable type: Binary variables

Measurement: “Please select the antidepressant that you have been prescribed from the list below: (Check more than one if you are taking more than one)”

Response options: Yes (TRUE), No (FALSE) for 21 antidepressant medication + 1 free entry field for participants to write in unlisted antidepressant medication type.

Variable name: 22 variables with med in front of medication name

### Selective Serotonin Reuptake Inhibitors (SSRIs)

- Citalopram
- Escitalopram
- Fluoxetine
- Fluvoxamine
- Paroxetine
- Sertraline

### Serotonin and Norepinephrine Reuptake Inhibitors (SNRIs)

- Desvenlafaxine
- Duloxetine
- Levomilnacipran
- Venlafaxine

### Tricyclics

- Amitriptyline
- Clomipramine
- Doxepin
- Dosulepin
- Imipramine
- Trimipramine
- Amoxapine
- Despiramine
- Nortriptyline
- Protriptyline

### Atypical Antidepressants

- Agomelatine
- Mirtazapine

## Antidepressant Medication Dosage

*Asked for the antidepressant arm only

Variable type: Binary variables

Measurement: “Please select the statement that best applies to you”

Response options: Yes (TRUE), No (FALSE) for 3 binary variables including 1) taking exact dosage, 2) taking less dosage, 3) taking more dosage

Variable name: Taking_Exact_Dosage, Taking_Less_Dosage, Taking_More_Dosage

## Antidepressant Medication Side Effects

*Asked for the antidepressant arm only

### Presence of antidepressant side effects

Variable type: Binary variable

Measurement: “Have you been bothered by any side-effects?”

Response options: Yes (TRUE), No (FALSE)

Variable name: SideEff

### Types of antidepressant side effects

Variable type: Binary variable

Measurement: “Please tell us the side-effect(s) you have been most bothered by:”

Response options: Yes (TRUE), No (FALSE) for 10 side effects + 1 free entry field for other unlisted antidepressant side effects.

Variable name: 11 variables with SideEff in front of side effect type

- Difficulty thinking or remembering (SideEff_CogDiff)
- Day-time sleepiness (SideEff_DaySl)
- Gastrointestinal symptoms e.g. nausea, diarrhea, constipation (SideEff_Gastro)
- Migraines/Headaches (SideEff_MigHe)
- Muscle/joint aches (SideEff_MusJoi)
- Night-time sleep disturbance (SideEff_NightSl)
- Personality changes, such as appearing flat, or without emotion (SideEff_PersCh)
- Sexual problems (SideEff_SexPr)
- Suicidal thoughts (SideEff_SuicTh)
- Weight Gain (SideEff_WeightG)
- Other side-effect(s) not listed above (SideEff_Other)

## Treatment Adherence

### Treatment adherence (antidepressant)

Variable type: Binary variable

Measurement: “Are you still taking an antidepressant medication?”

Response options: Yes (TRUE), No (FALSE)

Variable name: Taking_Antidepressant

### Treatment adherence for (iCBT)

Variable type: Binary variable

Measurement: “Are you still taking an online CBT course?”

Response options: Yes (TRUE), No (FALSE)

Variable name: Taking_CBT

### Treatment non-adherence reasons (antidepressant)

Variable type: Binary variables

Measurement: “Please tell us why you stopped taking the antidepressant:”

Response options: Yes (TRUE), No (FALSE) for 4 binary variables + 1 free entry field for other unlisted treatment non-adherence reasons

Variable name: 5 variables with non_adhere in front of the treatment non-adherence reason

- My mental health symptom got worse (nonadhere_gotworse)
- The medication was not making me feel better (nonadhere_nothelpful)
- Side effects were too strong (nonadhere_strongsideeff)
- Other reason (nonadhere_other)
- Other reason (free entry field; nonadhere_otherreason)

### Treatment non-adherence reasons (iCBT)

Variable type: Binary variables

Measurement: “Please tell us why you stopped:”

Response options: Yes (TRUE), No (FALSE) for 4 binary variables + 1 free entry field for other unlisted treatment non-adherence reasons

Variable name: 5 variables with non_adhere in front of the treatment non-adherence reason

- My mental health symptom got worse (nonadhere_gotworse)
- The CBT was not making me feel better (nonadhere_nothelpful)
- I found the CBT too difficult (nonadhere_toodifficult)
- Other reason (nonadhere_other)
- Other reason (free entry field; nonadhere_otherreason)

## Extra Treatment Information

Variable type: Binary variable

Measurement: “Is there any other information you would like us to know that we have not asked?”

Response options: Yes (TRUE), No (FALSE) + 1 free entry field for entering extra information (conditional on TRUE)

Variable name: Extra_WCI_Info (TRUE/FALSE), ExtraInfo (free filling box)

## Concurrent Medication Treatment

Variable type: Binary variable

Measurement: 2 separate questions asked for each treatment arm at 3 weekly check-in timepoints. “Have you started taking any other medications?” (antidepressant) and “Are you currently taking any medication for your mental health?” (iCBT)

Response options: Yes (TRUE), No (FALSE)

Variable name: Taking_OtherMed

### Concurrent medication treatment type (antidepressant)*

*Conditional on TRUE in Taking_OtherMed (antidepressant)

Variable type: Categorical string variables

Measurement: “Please provide details about the medication(s):”

Response options: 3 free entry fields. It was not compulsory for participants to provide this information.

Variable name: OtherMed_1, OtherMed_2, OtherMed_3

### Concurrent medication treatment dosage (antidepressant)*

*Conditional on TRUE in Taking_OtherMed (antidepressant)

Variable type: Continuous discrete variable

Measurement: “Please provide details about the medication(s):”

Response options: 3 free entry fields. It was not compulsory for participants to provide this information.

Variable name: OtherMedDose_1, OtherMedDose_2, OtherMedDose_3

### Concurrent medication treatment type (iCBT)*

*Conditional on TRUE in Taking_OtherMed (iCBT)

Variable type: Binary variables

Measurement: “Please select the antidepressant that you have been prescribed from the list below: (Check more than one if you are taking more than one)”

Response options: Yes (TRUE), No (FALSE) for 21 antidepressant medication + 1 free entry field for other unlisted antidepressant medication type. It was not compulsory for participants to provide this information.

Variable name: 22 variables with med in front of medication name (see Antidepressant Medication Type)

## Concurrent Psychotherapy Treatment

### Concurrent psychotherapy treatment (baseline)

Variable type: Binary variable

Measurement: Are you currently receiving regular psychological therapy?

Response options: Yes (1), No (0)

Variable name: TFLTE_PTcurrent

### Types of concurrent psychotherapy treatment (baseline)*

*Conditional on TRUE in TFLTE_PTcurrent

Variable type: Binary variables

Measurement: “Please select the type of psychological therapy:”

Response options: Yes (TRUE), No (FALSE) for 10 psychotherapy interventions. It was not compulsory for participants to provide this information.

Variable name: 10 variables with PT in front of psychotherapy short-handed name (e.g., TFLTE_PTcurrent_CBT)

- Behavioural therapy (TFLTE_PTcurrent_BT)
- Cognitive Behavioural Therapy (TFLTE_PTcurrent_CBT)
- Counselling (TFLTE_PTcurrent_Counsig)
- Cognitive Therapy (TFLTE_PTcurrent_CT)
- Family Therapy (TFLTE_PTcurrent_FT)
- Interpersonal Therapy (TFLTE_PTcurrent_IPT)
- Mindfulness (TFLTE_PTcurrent_Mindflns)
- Other (TFLTE_PTcurrent_Other)
- Psychoanalytic (TFLTE_PTcurrent_Psychoan)
- Psychodynamic (TFLTE_PTcurrent_Psychodyn)

### Concurrent psychotherapy treatment (weekly)

Variable type: Binary variable

Measurement: 2 separate questions asked for each treatment arm at 3 weekly check-in timepoints. “Are you currently receiving regular psychological therapy?” (antidepressant) and “Have you started any other form of psychological therapy?” (iCBT)

Response options: Yes (TRUE), No (FALSE)

Variable name: PT_WCI

### Concurrent psychotherapy treatment type (weekly)*

*Conditional on TRUE in concurrent psychotherapy treatment (weekly)

Variable type: Binary variables

Measurement: “Please select the type of psychological therapy:”

Response options: Yes (TRUE), No (FALSE) for 10 psychotherapy interventions. It was not compulsory for participants to provide this information.

Variable name: 10 variables with PT in front of psychotherapy short-handed name

- Behavioural therapy (PT_WCI_BT)
- Cognitive Behavioural Therapy (PT_WCI_CBT)
- Counselling (PT_WCI_Counsig)
- Cognitive Therapy (PT_WCI_CT)
- Family Therapy (PT_WCI_FT)
- Interpersonal Therapy (PT_WCI_IPT)
- Mindfulness (PT_WCI_ Mindflns)
- Other (PT_WCI_Other)
- Psychoanalytic (PT_WCI_Psychoan)
- Psychodynamic (PT_WCI_Psychodyn)

## iCBT Treatment Engagement

*Measurement is taken objectively as users engage with the iCBT platform

### Total time spent (seconds)

Variable type: Continuous discrete variable

Measurement: The combination of the time spent in each session (in seconds) from the first to the last log-in. Interactions lasting longer than 30 minutes are automatically counted as 1 minute, to avoid counting long idle periods when the program is open toward the total count.

Variable name: total_time_spent_(seconds)

### Number of sessions

Variable type: Continuous discrete variable

Measurement: The number of times (logins) the user accessed the program. If a specific session has inactivity periods longer than 30 minutes, the next moment of activity will count as a new session.

Variable name: number_of_sessions

### Average session length (seconds)

Variable type: Continuous variable

Measurement: Dividing the total time on the platform by the number of sessions.

Variable name: avg_session_length_(seconds)

### Number of activities

Variable type: Continuous variable

Measurement: Every instance a user interacted actively with the platform, e.g., completed a journal entry, used an interactive tool, downloaded, or played relaxation audios. Participants were able to use these activities as many times as they wished.

Variable name: num_activities

### Number of modules in program

Variable type: Continuous discrete ariable

Measurement: The total number of modules available to the user specific to the iCBT program assigned to them.

Variable name: no_of_modules_in_program

### Modules completed

Variable type: Continuous discrete variable

Measurement: The number of modules out of the total number of modules available in the program that the user completed.

Variable name: num_activities

### Percentage of program viewed

Variable type: Continuous variable

Measurement: Ranging from 0-1, this metric captures the proportion of the program that is completed by the user.

Variable name: percentage_programme_viewed

### Activities per session

Variable type: Continuous variable

Measurement: Dividing the total number of activities completed by the total number of sessions.

Variable name: activities_per_session

### Modules per session

Variable type: Continuous variable

Measurement: Dividing the total number of modules completed by the total number of sessions.

Variable name: modules_per_session

### Number of journal entries

Variable type: Continuous discrete variable

Measurement: The total number of journal entries submitted by the user on the platform.

Variable name: number_journal_entries

### Length of journal entries

Variable type: Continuous discrete variable

Measurement: The total number of characters used in the total number of journal entries submitted by the user on the platform.

Variable name: length_journal_entries

### Supporter Experience

Variable type: Continuous discrete variable

Measurement: The total number of reviews completed by the clinician prior to their assignment to the user. i.e. the level of experience of the clinician managing the client.

Variable name: supporter_experience

### Number of reviews

Variable type: Continuous discrete variable

Measurement: The total number of reviews sent from the assigned clinician to the user so to encourage use of the platform while monitoring and providing feedback about the progress from the last review.

Variable name: num_reviews

### Length of reviews

Variable type: Continuous discrete variable

Measurement: The sum of the total number of characters used in all reviews written by the clinician for the user.

Variable name: length_of_reviews

### Average review length

Variable type: Continuous discrete variable

Measurement: Dividing the length of reviews by the number of reviews.

Variable name: avg_review_length

### Reviews fit versus template

Variable type: Continuous variable

Measurement: Ranging from 0-1, this is a percentage showing how similar the review text is to a set of templates that are used by the service. A lower value means the message was more customised, and a higher value means the closer / more similar to the templates.

Variable name: reviews_fit_vs_template

### Number of review notes

Variable type: Continuous discrete variable

Measurement: The number of replies that the user left for their clinician after a review.

Variable name: number_reviewnotes

### Review note length

Variable type: Continuous discrete variable

Measurement: The total number of characters in the review notes left by the user for their clinician after a review.

Variable name: note_length

### Baseline Depression Score

Variable type: Continuous discrete variable

Measurement: Patient Health Questionnaire (PHQ-9) (18)

Response options: A 4 Likert scale for 9 items: Not at all (0), Several days (2), More than half the days (3), Nearly every day (4). A total score is yielded by summing item responses. The higher the score, the more depressed the individual. This measure is taken at baseline (i.e., when participants first logged onto the platform).

Variable name: phq_time1

### Baseline Anxiety Score

Variable type: Continuous discrete variable

Measurement: Generalised Anxiety Disorder Assessment (GAD-7) (19)

Response options: A 4 Likert scale for 7 items: Not at all (0), Several days (2), More than half the days (3), Nearly every day (4). A total score is yielded by summing item responses. The higher the score, the more anxious the individual. This measure is taken at baseline (i.e., when participants first logged onto the platform).

Variable name: gad7_time1

### Number of tools used

Variable type: Continuous discrete variable

Measurement: The total number of tools used by the user on the platform. Examples include Mood Monitor and Worry Tree.

Variable name: num_tools_used

### Modules viewed

Variable type: Continuous discrete variable

Measurement: The total number of modules within a specific assigned program that were viewed by users on the platform. Partially viewed modules as well as fully viewed modules contribute to this metric.

Variable name: modules_viewed

### Mode of Engagement

Variable type: Continuous variable

Measurement: Ranging from 0-1, the percentage the user spent on each digital application to access the platform. Currently, iCBT can be accessed through the phone (mobile), the tablet, the computer (pc), or the SilverCloud app.

Variable name: mobile, tablet, pc, app

### Site

Variable type: Categorical variable

Measurement: The mental health service the user registered with to access their iCBT treatment. Participants in the study either registered with the mental health charity Aware in Dublin, Ireland or the Talking Therapies services in Berkshire, London, UK.

Variable name: aware, Berkshire

### SilverCloud Program Name

Variable type: Categorical variable

Measurement: The assigned iCBT treatment program to the user by the mental health service based on patients’ mental health presentations. SilverCloud has over 40 programs across the spectrum of mental health available to their user. Participants registered with the mental health charity Aware in Dublin, Ireland were only assigned the Life Skills Online program. Below includes a non-exhaustive list of SilverCloud programs available to participants in our study.

Variable name: silvercloud_program_name

- Space from Depression
- Life Skills Online
- Space from GAD
- Space from Depression & Anxiety
- Space from Anxiety
- Space from Stress
- Space from Social Anxiety
- Space for Perinatal Wellbeing
- Space for Resilience
- Space in Chronic Pain from Depression & Anxiety
- Space from OCD
- Space from Health Anxiety
- Space from Phobia
- Space from Panic
- Space for Sleep
- Space in Lung Conditions from Depression & Anxiety
- Space in Diabetes from Depression & Anxiety

# Cognitive Performance

## Perceptual Decision-Making Task

Variable type: Cognitive test data, numerous derived measures

Measurement: Dot discrimination task based on (20). A perceptual decision-making task which dissociates between decision-formation and two components of metacognitive evaluation – confidence bias (subjective confidence regardless of fluctuations owing to performance) and metacognitive efficiency (the sensitivity of one’s confidence reports to correct/incorrect judgements). Gamified as a task where participants guess which sunflower has more seeds (Figure eB1).

Response options: Left, Right (select sides with more dots). Confidence ratings on 6-point scale from guessing to certain.

Variable name: Metacognitive bias, metacognitive efficiency, reaction time, accuracy, stimulus intensity deviations, non-decision time, decision threshold, baseline drift rate, effect of dot difference on drift rate


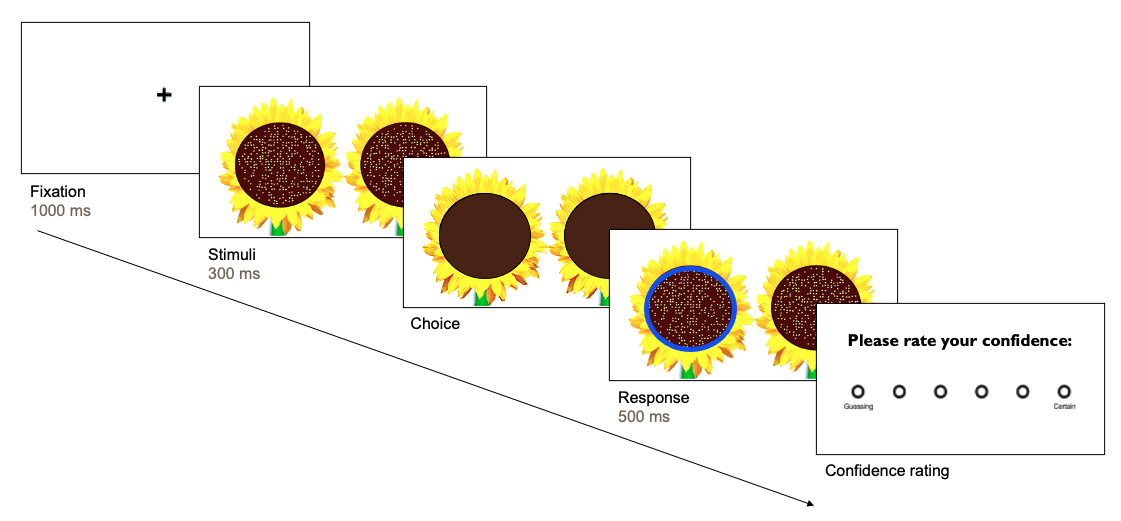


Figure eB1. Dot discrimination task for assessing metacognitive evaluation.

## Two-Step Reinforcement-Learning Task

Variable type: Cognitive test data, numerous derived measures

Measurement: Two-step reinforcement-learning task (21,22). A reinforcement learning task where two sequential decisions are made on each trial with a goal of maximising reward that is probabilistically associated with stimuli in the second stage choice. It allows derivation of model-based and model-free learning estimates per participant (Figure eB2).

Response options: Left, Right (select planets). Left, Right (select aliens).

Variable name: Model-based learning, Model-free learning, Reaction Time, RT sensitivity to Transition Structure, Choice stochasticity, Learning rate, Choice stickiness


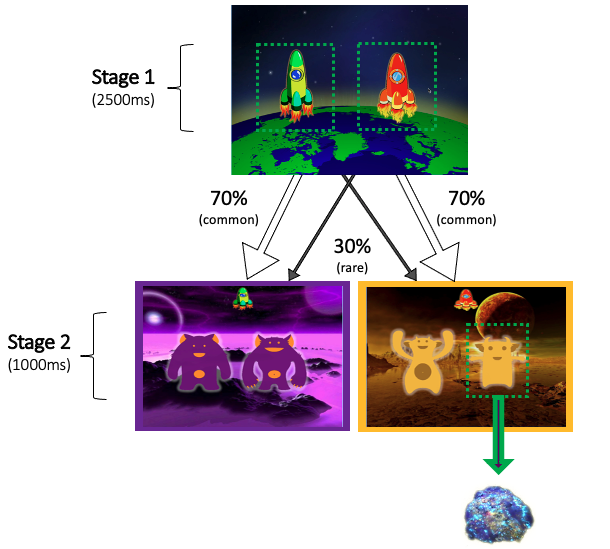


Figure eB2. Reinforcement learning task (N=200 trials) for assessing goal-directed learning.

## Learning Under Volatility Task

Variable type: Cognitive test data, numerous derived measures

Measurement: An gamified version of an aversive learning task (23) was used to measure how well subjects learning rates adjust to environmental volatility (Figure eB3).

Response options: Left, Right (select urchin).

Variable name: Reaction Time, Choice stochasticity, Learning rate, Learning Rate Adjustment based on Volatility, Choice stickiness, Risk preference

Figure eB3. Aversive learning task for assessing relative learning rate.

## Abstract Reasoning Test^[[7]](#footnote-8)^

Variable type: Cognitive test data, numerous derived measures

Measurement: Computerized Adaptive Test (CAT) based on a bank of 26 items similar to that used in the Raven’s Standard Progressive Matrices (24) previously operationalised in a prior study (25) (Figure eB4).

Response options: Mouse selection, 1 of 8 options

Variable name: IQ (adaptive generated theta value); number of correct responses, reaction time.


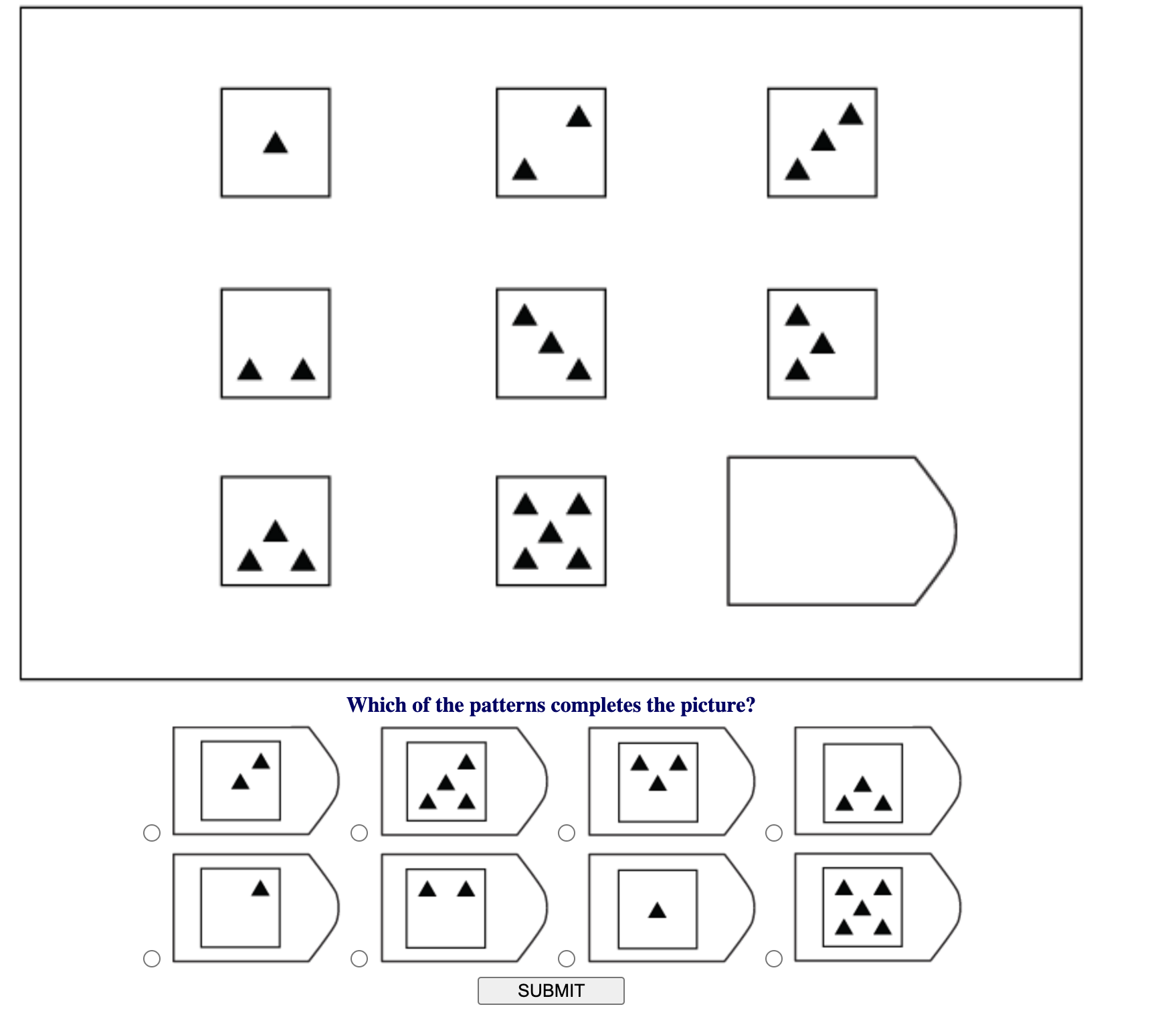


Figure eB4. An example of trial item of the IQ Computerized Adaptive Test in measuring abstract reasoning

# Data Quality Probes

## Distraction Probe

Variable type: Binary variables

Measurement: “Were you distracted by any of the following while you completed the study?”

Response options: Self-report checkboxes (TRUE for yes and FALSE for no) for the following distraction types: TV, Phone (e.g., phone call/text message/social media), Something on the computer that you used for the study, interruptions from family/friends, Pet, Knock on the door, background noises, other (free text). If participants did not select any of these options, their response was coded as “not distracted”. If they selected any of the above responses, they were coded as ‘distracted’

## Substance Use Probe

Variable type: Binary variables

Measurement: “Did you consume any of the following substances within 5 hours of starting the study?”

Response options: Self-report checkboxes (TRUE for yes and FALSE for no) for the following substance types: Alcohol, Marijuana, Ecstasy/MDMA, Stimulant drugs (cocaine/amphetamine), Opiates (e.g. codeine, oxycodone, heroin), sedatives/tranquilisers (e.g., Valium/Xanax), Other (free text). If participants did not select any of these options, they response was coded as “no substance use”. If participants selected any of the specified substances above, they were coded ‘substance use’. In contrast to the distraction probe, if participants selected ‘Other’, they were coded as ‘no substance use’. This is because based on the free text data, participants selected this option if they had taken coffee or prescription medicine, which we do not consider problematic substance use for the purposes of this item.

## Attention Check OCI

Variable type: Data quality probe

Measurement: “If you are paying attention, select ‘a little’ as your response”

Response options: A 5-point Likert scale: Not at all (0), A little (1), Moderately (2), A lot (3), Extremely (4).

Variable name: OCItrap

## Attention Check WSAS

Variable type: Data quality probe

Measurement: “If you are paying attention to these questions, please select "Not at all" as your answer”

Response options: A 8-point Likert scale for 5 items: Not at all (0), Slightly (2), Definitely (4), Markedly (6), Very severely (8).

Variable name: WSAScatch

# References

1. Adler NE, Epel ES, Castellazzo G, Ickovics JR. Relationship of subjective and objective social status with psychological and physiological functioning: preliminary data in healthy white women. Health Psychol. 2000;19(6):586–92.

2. Linn BS, Linn MW, Gurel LE. Cumulative illness rating scale. J Am Geriatr Soc. 1968;16(5):622-6.

3. Kroenke K, Spitzer RL, Williams JB. The PHQ-15: validity of a new measure for evaluating the severity of somatic symptoms. Psychosom Med. 2002;64(2):258-66.

4. Holmes TH, Rahe RH. The social readjustment rating scale. J Psychosom Res. 1967;11(2):213-18

5. Pennebaker JW, Susman JR. Disclosure of traumas and psychosomatic processes. Soc Sci Med. 1988;26(3):327–32.

6. Zimet GD, Dahlem NW, Zimet SG, Farley GK. The multidimensional scale of perceived social support. J Pers Assess. 1988;52(1):30-41.

7. Cohen S, Kamarck T, Mermelstein R. A global measure of perceived stress. J Health Soc Behav. 1983:385-96.

8. Chekroud AM, Zotti RJ, Shehzad Z, Gueorguieva R, Johnson MK, Trivedi MH, Cannon TD, Krystal JH, Corlett PR. Cross-trial prediction of treatment outcome in depression: a machine learning approach. Lancet Psychiatry. 2016;3(3):243-50.

9. Marin RS, Biedrzycki RC, Firinciogullari S. Reliability and validity of the apathy evaluation scale. Psychiatry Res. 1991;38(2):143–62.

10. Patton JH, Stanford MS, Barratt ES. Factor structure of the Barratt impulsiveness scale. J Clin Psychol. 1995;51(6):768-74.

11. Garner DM, Olmsted MP, Bohr Y, Garfinkel PE. The eating attitudes test: psychometric features and clinical correlates. Psychol Med. 1982;12(4):871-8.

12. Liebowitz MR. Social Phobia. Mod Probl Pharmacopsychiatry; 1987;22:141-173.

13. Mason O, Linney Y, Claridge G. Short scales for measuring schizotypy. Schizophr Res. 2005;78(2-3):293-6.

14. Zung WW. A self-rating depression scale. Arch Gen Psychiatry. 1965;12(1):63-70.

15. Spielberger CD, Gorsuch RL, Lushene R, Vagg PR, Jacobs GA. Manual for the State-Trait Anxiety Inventory. Consulting Psychologists Press; 1983.

16. Foa EB, Huppert JD, Leiberg S, Langner R, Kichic R, Hajcak G, Salkovskis PM. The Obsessive-Compulsive Inventory: development and validation of a short version. Psychol Assess. 2002;14(4):485.

17. Rush AJ, Trivedi MH, Ibrahim HM, Carmody TJ, Arnow B, Klein DN, Markowitz JC, Ninan PT, Kornstein S, Manber R, Thase ME. The 16-Item Quick Inventory of Depressive Symptomatology (QIDS), clinician rating (QIDS-C), and self-report (QIDS-SR): a psychometric evaluation in patients with chronic major depression. Biol Psychiatry. 2003;54(5):573-83.

18. Kroenke K, Spitzer RL, Williams JBW. The PHQ‐9: validity of a brief depression severity measure. J Gen Intern Med. 2001;16(9):606–13.

19. Spitzer RL, Kroenke K, Williams JBW, Löwe B. A Brief Measure for Assessing Generalized Anxiety Disorder. Arch Intern Med. 2006;166(10):1092.

20. Rouault M, Seow T, Gillan CM, Fleming SM. Psychiatric symptom dimensions are associated with dissociable shifts in metacognition but not task performance. Biol Psychiatry. 2018;84(6):443-51.

21. Daw ND, Gershman SJ, Seymour B, Dayan P, Dolan RJ. Model-based influences on humans' choices and striatal prediction errors. Neuron. 2011;69(6):1204-15.

22. Decker JH, Otto AR, Daw ND, Hartley CA. From creatures of habit to goal-directed learners: Tracking the developmental emergence of model-based reinforcement learning. Psychol Sci. 2016;27(6):848-58.

23. Behrens TEJ, Woolrich MW, Walton ME, Rushworth MFS. Learning the value of information in an uncertain world. Nat Neurosci. 2007;10(9):1214–21.

24. Raven J. The raven’s progressive matrices: Change and stability over culture and time. Cogn Psychol. 2000;41(1):1–48.

25. Gillan CM, Kosinski M, Whelan R, Phelps EA, Daw ND. Characterizing a psychiatric symptom dimension related to deficits in goaldirected control. Elife. 2016;5:1–24.

1. Due to non-normality of the variable, the responses were binned and converted into a categorical variable 3 distinct groups: 1) <2 lifetime episodes, 2) 2-5 lifetime episodes, and 3) >5 lifetime episodes. [↑](#footnote-ref-2)
2. Due to technical glitch in the survey, a small number of participants are missing the specific day / month of the year of onset. For instances missing the day (DD), the first day of the month/year provided was designated as the day of the date (i.e., 1^st^ of the month). For instances missing the day and the month (DD/MM), the first day and first month of the year provided was designated as the day and month of the date (i.e., 1^st^ January). [↑](#footnote-ref-3)
3. The AES score labels were abbreviated in our study during administration of the measure. The response options traditionally read (1) Not at all characteristic, (2) Slightly characteristic, (3) Somewhat characteristic, (4) Very characteristic. We simply used (1) Not at all, (2) Slightly, (3) Somewhat, (4) A lot. [↑](#footnote-ref-4)
4. In the original version of the scale, participants answered all 24 items twice each time pertaining to how anxious or fearful they feel in the situation respectively. Prior work has shown these correlate with one another near perfectly, so we merged the response option to reduce redundancy; participants answered all 24 items just once with regards to how anxious OR fearful they feel in the situation. [↑](#footnote-ref-5)
5. There were errors associated with the administration of the STAI-T measure. 1) The opening instructions were taken from the SDS measure rather than the STAI measure. Thus, instead of asking subjects to rate how they ‘generally feel”, they were instead asked to rate the response that “best describes how often you felt or behaved this way during the past several days.” 2) The STAI response options were taken from the STAI State form (“not at all”, “somewhat”, moderately so”, very much so”) instead of STAI Trait Form (“almost never”, “sometimes”, “often”, almost always”). [↑](#footnote-ref-6)
6. The last item of the QIDS was abbreviated from “I think of suicide or death several times a day in some detail, or I have made specific plans for suicide or have actually tried to take my life” to (“I think of suicide or death several times a day in some detail, or have actually tried to take my life. [↑](#footnote-ref-7)
7. Due to technical errors, the completion of the Abstract Reasoning Test was not required for participants to progress further with the study. As a result, data on this cognitive task were gathered on a subsample of participants in the study. [↑](#footnote-ref-8)
